# 3-Hydroxyphthalic Anhydride- Modified Rabbit Anti-PAP IgG as a Potential Bifunctional HIV-1 Entry Inhibitor

**DOI:** 10.3389/fmicb.2018.01330

**Published:** 2018-06-19

**Authors:** Xuanxuan Zhang, Jinquan Chen, Fei Yu, Chunyan Wang, Ruxia Ren, Qian Wang, Suiyi Tan, Shibo Jiang, Shuwen Liu, Lin Li

**Affiliations:** ^1^Guangdong Provincial Key Laboratory of New Drug Screening, Guangzhou Key Laboratory of Drug Research for Emerging Virus Prevention and Treatment, School of Pharmaceutical Sciences, Southern Medical University, Guangzhou, China; ^2^Jiangsu Food and Pharmaceutical Science College, Huai’an, China; ^3^Key Laboratory of Medical Molecular Virology of Ministries of Education and Health, School of Basic Medical Sciences and Shanghai Public Health Clinical Center, Fudan University, Shanghai, China; ^4^College of Life Sciences, Agricultural University of Hebei, Baoding, China; ^5^Center for Clinical Laboratory, Nanfang Hospital, Southern Medical University, Guangzhou, China; ^6^Lindsley F. Kimball Research Institute, New York Blood Center, New York, NY, United States

**Keywords:** HIV, 3-hydroxyphthalic anhydride-modified rabbit anti-PAP IgG (HP-API), prostatic acidic phosphatase (PAP), semen-derived enhancer of viral infection (SEVI), HIV entry inhibitor

## Abstract

Several studies have reported that amyloid fibrils in human semen formed from a naturally occurring peptide fragment of prostatic acidic phosphatase (PAP248-286), known as semen-derived enhancer of viral infection (SEVI), could dramatically enhance human immunodeficiency virus type 1 (HIV-1) infection. Accordingly, SEVI might serve as a novel target for new antiviral drugs or microbicide candidates for the prevention of sexually transmitted HIV. Theoretically, a special anti-PAP or anti-SEVI antibody could reduce the enhancement of viral infection by blocking the binding of HIV and SEVI fibrils. Here, 3-hydroxyphthalic anhydride modified anti-PAP248-286 antibody, named HP-API, exhibited broad-spectrum and highly effective anti-HIV-1 activities on different subtypes and tropism. By using time-of-addition, cell–cell fusion and a single-cycle HIV-1 infection assays, we demonstrated that HP-API is an HIV-1 entry/fusion inhibitor. Mechanism studies suggest that HP-API inhibited HIV-1 entry/fusion by targeting both HIV-1 gp120 envelop and CD4 receptor on the host cell specifically. It is noteworthy that HP-API abrogated the formation of SEVI fibrils and partially interfered with SEVI-mediated enhancement of HIV-1 infection. Based on these findings, HP-API could be considered a bifunctional HIV-1 entry/fusion inhibitor with high potential.

## Introduction

Currently, the high risk of unprotected sexual intercourse among sexually active individuals is a major cause to HIV disease progression (UNAIDS, 2016). Continual failures of microbicide candidates in clinical trials imply that there should be some unknown reasons to affect their efficacy during application in human beings (Garg et al., 2009; Buckheit et al., 2010; Vanpouille et al., 2012; McGowan, 2014). Generally, a microbicide candidate in clinical trials is administrated by vagina or rectum. During sexual intercourse, the influence of female genital tract secretions and male semen in the host environment on the antiviral efficacy of those microbicides should not be ignored (Doncel et al., 2011; Sabatte et al., 2011; Saidi et al., 2012).

Notably, it has recently been reported that semen boosted HIV infectivity and impaired the anti-HIV efficacy of microbicide candidates used in clinical trials *in vitro* (Zirafi et al., 2014). Some endogenous amyloid aggregates have been detected in healthy human semen samples (French et al., 2014; Usmani et al., 2014), which partially consist of prostatic acidic phosphatase (PAP) fragments that increase HIV infectivity by trapping viral particles (Roan et al., 2011; Arnold et al., 2012). Semen-derived enhancer of viral infection (SEVI) is the first such discovered endogenous amyloid aggregate, which is formed by a naturally occurring peptide fragment (the amino acid residues 248–286 of PAP) (Munch et al., 2007). Some researchers have confirmed that SEVI fibrils could assemble HIV virions by their cationic property and facilitate HIV attachment to the surface of target cells (Roan et al., 2009). Our previous study showed that SEVI fibrils might be one of the risk factors causing for those polyanionic microbicides to fail in clinical trials (Tan et al., 2013; Chen et al., 2015). Therefore, SEVI fibrils might play a critical role and serve as a novel target in identifying the best microbicide candidates for the prevention of sexually transmitted HIV.

Theoretically, a molecule may reduce the enhancement of viral infection by blocking the binding of HIV to seminal amyloid fibrils or preventing the formation of seminal amyloid fibrils (Castellano et al., 2015a; Lump et al., 2015). Microbicide candidates targeting the host’s proteins will be fundamentally different from the traditional microbicides targeting the virus itself. A specific anti-PAP or anti-SEVI antibody may be a good choice to inhibit the enhancement of viral infection caused by SEVI fibrils through binding to peptide PAP248-286 or SEVI fibrils directly.

Based on this tentative idea, we immunized normal rabbits with the peptide PAP248-286 and then purified the rabbit anti-PAP IgG (API) from the rabbit antisera. Although API binds to both PAP248-286 and SEVI fibrils, it showed no significant anti-HIV activities *in vitro*. We previously reported that an inactive protein might become an effective antiviral compound by anhydride modification on the site-specific amino acids (Li et al., 2010; Li L. et al., 2013; Li M. et al., 2013). Thus, we reasoned that the modification of API using a suitable anhydride might transform it into a potential bifunctional microbicide candidate with both anti-HIV and anti-SEVI activity. Here, we modified API with 3-hydroxyphthalic anhydride (HP), prepared 3-hydroxyphthalic anhydride-modified rabbit anti-PAP IgG (HP-API). We intended to investigate the anti-HIV efficacies of HP-API and to study whether the antiviral activities were related to interference with the SEVI-mediated enhancement of HIV-1 infectivity.

## Materials and Methods

### Reagents

Peptide PAP248-286 was synthesized using a standard solid- phase 9-fluorenylmethoxy carbonyl (Fmoc) method at GL Biochem Ltd. (Shanghai, China) and were purified by high-performance liquid chromatography (HPLC). HP, polyethyleneimine (PEI), biotin goat-anti-rabbit IgG, bovine serum albumin (BSA), 3-(4,5-dimethylthiazol-2-yl)-2,5-diphenyltetrazolium bromide (MTT), dimethyl sulfoxide (DMSO), thioflavin T (ThT), Congo Red Kits, biotinylated goat anti-rabbit IgG and FITC-goat-anti-rabbit-IgG were purchased from Sigma-Aldrich (St. Louis, MO, United States). The recombinant human CD4 protein and HIV-1_JR-FL_ gp120 were purchased from Sino Biological (Wayne, PA, United States).

The pNL4-3E-R-Luc plasmid, HIV-1 and VSV-G Env-encoding plasmids, the peGFP-Vpr plasmid, MT-2 cells, TZM-bl cells, CHO-WT cells, U87-CD4-CCR5 cells, U87-CD4-CXCR4 cells, HIV-1IIIB-infected H9 cells (H9/HIV-1IIIB), laboratory-adapted and primary HIV-1 strains, HL2/3 cells, HeLa cells, HeLa-CD4-LTR-β-gal cells, T20, AZT, AMD3100, Maraviroc, anti-p24 monoclonal antibody (183-12H-5C) and HIV immunoglobulin (HIV-IgG) were obtained from the National Institutes of Health AIDS Research and Reference Reagent Program. GHOST (3) Hi-5, HEK-293T cells and VK2/E6E7 cells were purchased from ATCC (Manassas, VA, United States). Plasmids encoding CXCR4-tropic HIV-1NL4-3, CCR5-tropic HIV-1SF-162, and dual-tropic HIV-181A and NL4-3 infectious clones were kindly provided by Jan Münch of Ulm University (Ulm, Badenwürttemberg, Germany). ProteoStat Amyloid Plaque Detection Kits were purchased from Enzo Life Sciences (Plymouth Meeting, PA, United States). Peripheral blood mononuclear cells (PBMCs) were isolated from the blood of healthy donors at the Nanfang Hospital by standard density gradient centrifugation by using Histopaque-1077 (Sigma, St. Louis, MO, United States).

### Preparation of API and HP-API

After immunizing rabbits by PAP248-286 using conventional methods, the blood sera of rabbits containing specific polyclonal antibodies against PAP248-286 were collected by AbMax Biotechnology (Beijing, China). The rabbit anti-PAP IgG (API) was purified from the antisera by Protein A-Sepharose 4 Fast Flow column (Amersham Biosciences, Piscataway, NJ, United States). The concentration of API was detected by the BCA Protein Assay Reagent Kit (Pierce, Rockford, IN, United States). Furthermore, API was modified by HP as previously described. Briefly, saturated HP (1.19 M in DMSO) was divided into five aliquots, and then each portion was added into API solution [18.75 μM in 0.1 M phosphate buffer saline (PBS, pH 7.4)] at 12 min intervals. The final concentration of HP is 60 mM and pH value is 8.5. The mixture was kept for another 1 h at room temperature (RT), extensively dialyzed against PBS, and filtered through 0.45 μm syringe filters (Gelman Sciences, Ann Arbor, MI, United States).

### Determination of the Binding of HP-API to PAP248-286 or SEVI Amyloid Fibrils

The lyophilized PAP248-286 (>95% purity) was dissolved in PBS at a concentration of 3 mg/ml. SEVI fibrils were generated by incubating PAP248-286 with agitation at 1,400 rpm at 37°C for 48 h in an Eppendorf Thermomixer (Hamburg, Germany) as described previously. The binding of HP-API to PAP248-286 or SEVI fibrils were detected by ELISA and isothermal titration calorimetry (ITC).

For ELISA, wells of 96-half well polystyrene plates (Costar; Corning, Inc., Corning, NY, United States) were coated with PAP248-286 or SEVI fibrils at graded concentrations in 0.85 M carbonate-bicarbonate buffer (pH 9.6) at 4°C overnight. After blocking with 3% BSA in PBS at 37°C for 1 h, 12.5 nM HP-API was added into the wells and incubated at 37°C for another 1 h. Then, biotin-labeled goat-anti-rabbit-IgG (Sigma), SA-HRP and TMB were added into the reaction system successively. 1 N H_2_SO_4_ was used to terminate the reaction. The absorbance at 450 nm was measured by an ELISA reader (Ultra 384; Tecan, Research Triangle Park, NC, United States). API and normal rabbit IgG were used as controls.

The binding of HP-API to PAP248-286 was further detected by ITC assay using a NANO ITC (TA Instruments, New Castle, DE, United States) (Xun et al., 2015). The reference cells and the sample cells were stirred at 300 rpm at 37°C. A series of small aliquots of PAP248-286 solution (2.5 μl, 200 μM) were injected into a sample cell containing 300 μl of HP-API solution (6 μM) at intervals of 2 min for 20 injections. Blank ITC experiments were applied to correct heat-of-dilution effects. The enthalpy changes (ΔH) and the binding constant (*K*_d_) of the interaction between HP-API and PAP248-286 in solution were calculated by a NANO ITC software. API was used as a control.

### Monitoring the Effects of HP-API on the Formation of SEVI Fibrils

The graded concentrations of HP-API (600, 300, 150, 75, and 0 nM) were agitated with peptide PAP248-286 (3 mg/ml) at 1,400 rpm at 37°C for 48 h using an Eppendorf Thermomixer. The mixed samples were collected at different time points following agitation (0, 8, 16, 24, and 48 h) and were measured by ThT fluorescence assay, Congo red staining and transmission electron microscopy (TEM) as previously described (Chen et al., 2015). For ThT fluorescence assay, 5 μl mixed samples were stained by 195 μl ThT working solution (50 μM in PBS). Fluorescence intensity was measured immediately by an RF-5301 PC spectrofluorophotometer (Shimadzu). The excitation and emission wavelength were 440 nm (5 nm bandwidth) and 482 nm (10 nm bandwidth), respectively. For the Congo red assay, 10 μl mixed samples were added to 200 μl of Congo red solution contained in a Congo Red Kit (Sigma). And then the systems were incubated at RT for 2 min. Then the mixtures were centrifuged at 12,000 rpm for 5 min and the red-dyed fiber precipitates were dissolved in 50 μl DMSO. The absorbance at 490/650 nm was measured by an ELISA reader. The effects of HP-API on SEVI amyloid fibrils formation were further measured by TEM. In brief, SEVI fibrils (3 mg/ml) in the presence or absence of HP-API (300 nM) at different agitated time points (0, 8, 16, 24, and 48 h) were obtained and then sixfold diluted in PBS. The diluted samples were first deposited on glow-discharged, carbon-coated grids for 2 min, and then were negatively stained with 2% phosphotungstic acid for another 2 min. The morphologies of those amyloid fibrils samples were visualized at 20,000 magnifications with H-7650 transmission electron microscope at an accelerating voltage of 80 kV (Hitachi Limited, Tokyo, Japan). API was used as a control.

### Analysis of the Secondary Structure of SEVI Fibrils by Circular Dichroism (CD) Spectroscopy

The secondary β-sheet structures of SEVI fibrils with or without HP-API at different agitated time points (0 and 48 h) were observed by CD spectroscopy in the far-UV spectral region between 198 and 260 nm using a Jasco 715 spectropolarimeter (Jasco Inc., Japan) equipped with thermostat-controlled cell housing and cells with a 1-mm path length. The samples containing 3 mg/ml SEVI fibrils with 300 nM HP-API were diluted 15 times in PBS. All CD spectra were collected at RT and corrected for solution baseline via the subtraction of the corresponding blank samples. The quantities of SEVI amyloid fibrils with different secondary structures were estimated from the molar residue ellipticity using Jasco software utilities as previously described (Chen et al., 2015). API was used as a control.

### Detecting the Effects of HP-API on SEVI-Mediated HIV Enhancement

The enhancing activities of SEVI fibrils in the presence or absence of HP-API was further detected by a viral infection assay (Chen et al., 2015). Three kinds of HIV-1 infectious clones were produced by transfecting HEK-293T cells with proviral DNA expression plasmids (CXCR4-tropic NL4-3, CCR5-tropic SF-162, and dual-tropic 81A and NL4-3) via PEI transfection as previously described. SEVI fibrils (3 mg/ml) with or without HP-API (300 nM) were first agitated to allow fibril formation and collected at interval agitated time points (0, 8, 16, 24, and 48 h) at 1,400 rpm at 37°C. All samples were centrifuged at 12,000 rpm for 5 min and discarded the supernatant to remove the remaining HP-API. The enhancing activities of SEVI fibrils on infection by HIV-1 infectious clones (HIV-1_NL4-3_, HIV-1_SF-162_, and HIV-1_81A and NL4-3_) were determined as previously described. In brief, 1 × 10^5^/ml TZM-bl cells were seeded into 96-well microtiter plates and incubated at 37°C overnight. 2 ng of p24 antigen of viruses were incubated with the diluted SEVI fibril samples (final concentration is 50 μg/ml) at RT for 10 min prior to the addition of the mixture to TZM-bl cells. After 3 h post-infection, the culture supernatants were discarded and the fresh mediums were added for avoiding cytotoxicity. At 72 h post-infection, the cells were collected and lysed with the lysing reagent included in the luciferase kit (Promega Corp., Madison, WI, United States). Aliquots of cell lysates were transferred to 96-well flat bottom luminometer plates (Costar) and added to luciferase substrate (Promega). And then the infectious activities of those fibril samples were measured in an Ultra 384 luminometer (Tecan). API was used as a control.

### The Effect of HP-API on the Binding Between HIV-1 Virions and SEVI Fibrils

The effects of HP-API on the binding of HIV-1 virions to SEVI fibrils were first detected by a virus pull-down assay (Chen et al., 2015). HIV-1 Env-pseudotyped viruses (HIV-1_JR-FL_), infectious clones (HIV-1_NL4-3_, HIV-1_SF-162_ and HIV-1_81A and NL4-3_) and a primary HIV-1 strain (93BR020) were used. Briefly, 200 μg/ml SEVI fibrils were mixed with an equal volume of HP-API at graded concentrations (2,000, 500, 125, and 0 nM) and incubated at 37°C for 30 min. Furthermore, 10 μl viruses (about 100 ng/ml p24) were incubated with the mixed samples at 37°C for another 30 min. Then, the mixtures were centrifuged at 5,000 rpm for 3 min to pellet the fibrils and bound virions. The pellets were dissolved in PBS and mixed with equal volumes of 5% Triton X-100 at 4°C overnight. The virus lysates in the pellets were assayed for p24 antigens by ELISA as described before. Equal volumes of viruses (10 μl) were added to the ELISA reaction system and were mixed with 10 μl 5% Triton X-100 for testing the total p24 antigens. The proportion of p24 antigens in the pellets were then calculated (Pellets p24/Total p24). API was used as a control.

### Assessment of HP-API Antiviral Activity in the Presence or Absence of Preformed SEVI Fibrils

The inhibitory activities of HP-API against three different laboratory-adapted and primary HIV-1 strains [HIV-1 IIIB (X4), Bal (R5) and 93BR020 (X4R5)] in the presence of preformed SEVI fibrils were detected, respectively (Xun et al., 2015). Briefly, 1 × 10^5^/ml TZM-bl cells were seeded and incubated at 37°C overnight. The SEVI fibrils produced as described above were centrifuged 12,000 rpm for 5 min, and then the pellets were dissolved in fresh medium (final concentration is 50 μg/ml). The resuspended SEVI fibrils were incubated with HP-API at graded concentrations at 37°C for 30 min. Then, the mixture further co-incubated with 2 ng of p24 of viruses at RT for 10 min prior to the addition of the mixture to TZM-bl cells. After 3 h post-infection, the culture supernatants were changed with fresh medium. At 72 h post-infection, the luciferase activity was measured. The EC_50_ values were calculated using the Calcusyn software, kindly provided by Dr. T. C. Chou at Sloan-Kettering Cancer Center (New York, NY, United States).

The antiviral activities of HP-API against infection by different laboratory-adapted HIV-1 strains (HIV-1_IIIB_ and HIV-1_Bal_), primary HIV-1 viruses and drug-resistant HIV-1 strains (including HIV entry inhibitors and reverse transcriptase inhibitors resistant strains) without SEVI fibrils were analyzed as described elsewhere (Li et al., 2010). T20 was used as a positive control. Briefly, the graded concentrations of HP-API were incubated with 100 TCID_50_ (50% tissue culture infective dose) of different HIV-1 strains at 37°C for 30 min. MT-2 cells were used as target cells and p24 antigen were measured by ELISA after 4 days post-infection for detecting the inhibition of infection by HIV-1_IIIB_ (X4 strain) and HIV-1 drug-resistant strains including T20 and A17-resistant strains. TZM-bl cells were used as target cells and the luciferase activities were measured at 72 h post-infection for detecting the inhibition of infection by HIV-1_Bal_ (R5 strain). PHA-stimulated PBMCs were selected as target cells and p24 antigen were measured by ELISA after 7 days post-infection for assessing the antiviral activities on HIV-1 primary strains. The EC_50_ values were calculated using the CalcuSyn software.

To further verify the possible target(s) of HP-API, the antiviral activity of HP-API against HIV-1_IIIB_ infection on being pre-incubated either cells or virus particles prior to infection were analyzed. Briefly, the graded concentrations of HP-API were pre-incubated with 100 TCID_50_ of HIV-1_IIIB_ or MT-2 cells at 37°C for 30 min, respectively. After 4 days infection, TZM-bl cells were used as target cells and the luciferase activities were measured for detecting the inhibition of infection. The EC_50_ values were calculated using the CalcuSyn software.

### Cytotoxicity of HP-API *in Vitro*

The *in vitro* cytotoxicities of HP-API on HIV target cells (MT-2, U87-CD4-CXCR4, and U87-CD4-CCR5) and the human vaginal epithelial cells (VK2/E6E7) were measured by MTT assay. Briefly, equal amounts of HP-API at graded concentrations with cells described above were co-cultured at 37°C for 3 days. Then, 10 μl of 5 mg/ml MTT solution was added to each well. After incubating at 37°C for 4 h, the supernatants were discarded gently and 100 μl of DMSO was added for 10 min. The absorbance at 570 nm was measured by an ELISA reader. API was used as a negative control. The 50% cytotoxicity concentrations (CC_50_) were calculated using the CalcuSyn software.

### Time-of-Addition Assay

A time-of-addition assay was performed as previously described (Li et al., 2010). HP-API, API and the corresponding control anti-HIV compounds were added to the infection systems at different post-infection (0, 0.5, 1, 2, 4, 6, and 8 h). The antiviral activities of those compounds on the infection by HIV-1_IIIB_ and HIV-1_Bal_ were determined as described above. A nucleoside reverse transcriptase inhibitor (NRTI), AZT, was used as a positive control for inhibiting both HIV-1_IIIB_ and HIV-1_Bal_ infection. A CXCR4 co-receptor inhibitor, AMD3100, was chose as a negative control for inhibiting HIV-1_IIIB_ infection. In addition, a CCR5 co-receptor inhibitor, Maraviroc, was selected as a negative control for inhibiting HIV-1_Bal_ infection.

### Measurement the Inhibition of HP-API on HIV-1-Mediated Cell–Cell Fusion

The HIV-1-mediated cell–cell fusion assay was measured by two different methods as previously described (Li et al., 2010). MT-2 cells expressing CD4 receptor and CXCR4 co-receptor were used as the target cells. HIV-1_IIIB_ infected H9 (H9/HIV-1_IIIB_) cells or CHO-WT cells expressing gp120/gp41 were used as the effector cells. Briefly, MT-2 cells were incubated with the effector cells in the absence or presence of HP-API at graded concentrations at 37°C for 2 h (Calcein-AM labeled H9/HIV-1_IIIB_ cells) or 48 h (CHO-WT cells). The fused and unfused Calcein-labeled cells or the syncytia were counted under an inverted fluorescence microscope (Zeiss, Germany) or an inverted microscope, respectively. Four fields per well were examined randomly. T20 was used as a positive control.

### Assessment of the Inhibition of HP-API on a Single-Cycle HIV-1 Infection

In order to characterize the antiviral activity of HP-API on a single-cycle HIV-1 infection, HIV-1 viruses pseudotyped with different HIV-1 envelope proteins in comparison to VSV-G pseudovirus and H5N1/TG pseudovirus were generated as described above (Li et al., 2010). Briefly, HEK-293T cells were cotransfected with pNL4-3E^-^R^-^Luc plasmid and HIV-1 Env-encoding plasmids derived from HIV-1_HXB2_ (X4 strain), HIV-1_JR-FL_ (R5 strain), VSV-G Env-encoding plasmid or H5N1/TG Env plasmids by PEI transfection reagent. The inhibitions of HP-API against HIV-1, VSV-G and H5N1/TG pseudotyped virus infection were detected as described previously. Briefly, 1 × 10^5^/ml U87-CD4-CXCR4 cells, U87-CD4-CCR5 cells or MDCK cells were seeded in a 96-well plate at 37°C overnight. Then, HP-API at graded concentrations was preincubated with 100 TCID_50_ HIV-1, VSV-G and H5N1/TG pseudotyped virus for 30 min at 37°C. And then, the mixtures were transferred to the wells with the target cells. At 48 h post-infection, the luciferase activity was detected as described above. T20 was used as a positive control.

### Analysis the Binding of HP-API to Cells Expressing HIV-1 Env or CD4 Receptor

The binding of HP-API to cells expressing HIV-1 Env or CD4 receptor was detected by ELISA, flow cytometric analysis and surface plasmon resonance (SPR) analysis.

For ELISA and flow cytometric analysis, Hela-CD4-LTR-β-gal cells that express CD4 receptor were used to analyze the binding of HP-API to CD4 receptor. HL2/3 cells expressing high levels of HIV-1 Env proteins were used to evaluate the binding of HP-API to HIV-1 Env. HeLa cells bearing neither HIV-1 Env nor CD4 were chose as a negative cell control. API was also used as a negative drug control (Li L. et al., 2013).

For ELISA, 2 × 10^5^/well Hela-CD4-LTR-β-gal or HL2/3 cells were seeded at 37°C overnight, followed by fixing with 4% formaldehyde at RT for 15 min. After three washes, wells were blocked by 1% dry fat-free milk at 37°C for 1 h. HP-API at graded concentration was added and incubated for another 1 h at 37°C. Biotin-labeled goat-anti-rabbit-IgG, SA-HRP and TMB were added successively. 1 N H_2_SO_4_ was used to terminate the reaction. The absorbance at OD_450_ was measured by an ELISA reader.

For flow cytometric analysis, 1 × 10^6^/well Hela-CD4-LTR-β-gal or HL2/3 cells were suspended in PBS containing 10% goat serum (PBS-GS) and incubated at 4°C for 1 h. 10 nM HP-API was added to cells and incubated at 4°C for 1 h. After three washes, FITC-goat-anti-rabbit-IgG was added and incubated at 4°C for another 1 h. Then, the cells were washed six times and resuspended in 500 μl of wash buffer, followed by flow cytometry analysis.

The affinity of binding of HP-API to HIV-1 gp120 or CD4 was measured using BIAcore T100 system (GE Healthcare, Sweden) as previously described (Avramis et al., 2009). Briefly, gp120 derived from HIV-1_JR-FL_ (20 μg/ml) or CD4 (6 μg/ml) was immobilized onto a CM5 sensor chip, HP-API or API (5.00, 2.50, 1.25, 0.63, 0.31, and 0.16 nM) was injected at a flow rate of 20 μl/min with a contact time of 2 min and a dissociation time of 2.5 min. The running buffer was water. The chip platform was regenerated with 10 mM HEPES, 150 mM NaCl, and 0.01% vol/vol Tween 20 (pH 7.4) and washed with the running buffer. A binding affinity (*K*_D_) value was calculated using BIAcore software (GE Healthcare).

## Results

### HP-API Bound to Both the Peptide Fragment PAP248-286 and SEVI Amyloid Fibrils

**FIGURE 1:**
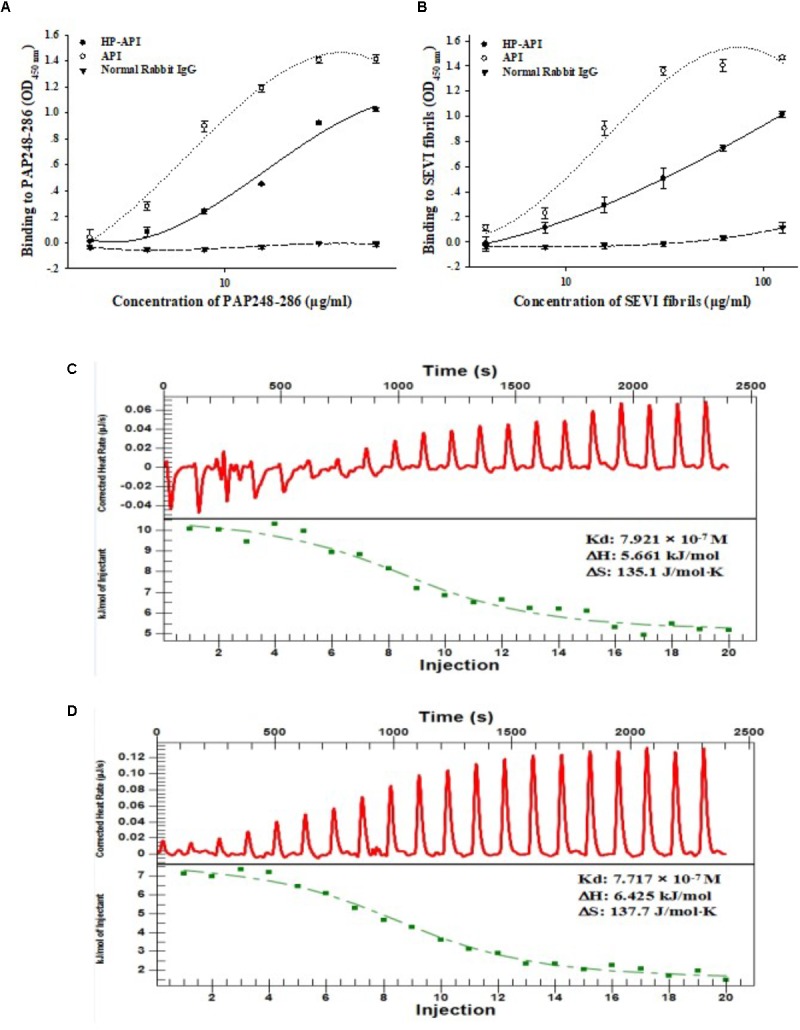
HP-API bound to both PAP248-286 and SEVI fibrils. Dose-dependent binding of HP-API to PAP248-286 **(A)** and SEVI fibrils **(B)** was detected by ELISA. API and normal rabbit IgG were used as controls. Data were presented in means ± SD. The binding of HP-API **(C)** and API **(D)** to PAP248-286 was analyzed by ITC assay.

A specific antibody of PAP248-286 or SEVI fibrils might be a good choice for blocking the SEVI-mediated enhancement of HIV infection due to its antigen-antibody specific effects. Here, we first prepared 3-hydroxyphthalic anhydride-modified rabbit anti-PAP IgG (HP-API) by chemical modification and further detected the binding of HP-API to the peptidic fragment of PAP248-286 or SEVI fibrils. Rabbit anti-PAP IgG (API) was used as a positive control antibody. Results showed that both HP-API and API could bind to the peptide PAP248-286 (**Figure 1A**) or SEVI fibrils (**Figure 1B**) in a dose-dependent manner by ELISA. Neither PAP248-286 nor SEVI fibrils could bind to the negative control antibody, a normal rabbit IgG (**Figures 1A,B**). Furthermore, ITC was conducted to obtain the thermodynamic parameters of the interaction between HP-API and PAP248-286 in solution. HP-API or API bound to PAP248-286 with a binding constant (*K*_d_) of 7.92 × 10^-7^ M (**Figure 1C**) or 7.72 × 10^-7^ M (**Figure 1D**), respectively, which indicated that both HP-API and API can bind to PAP248-286 with high affinity.

### HP-API Inhibited the Formation of SEVI Fibrils

**FIGURE 2:**
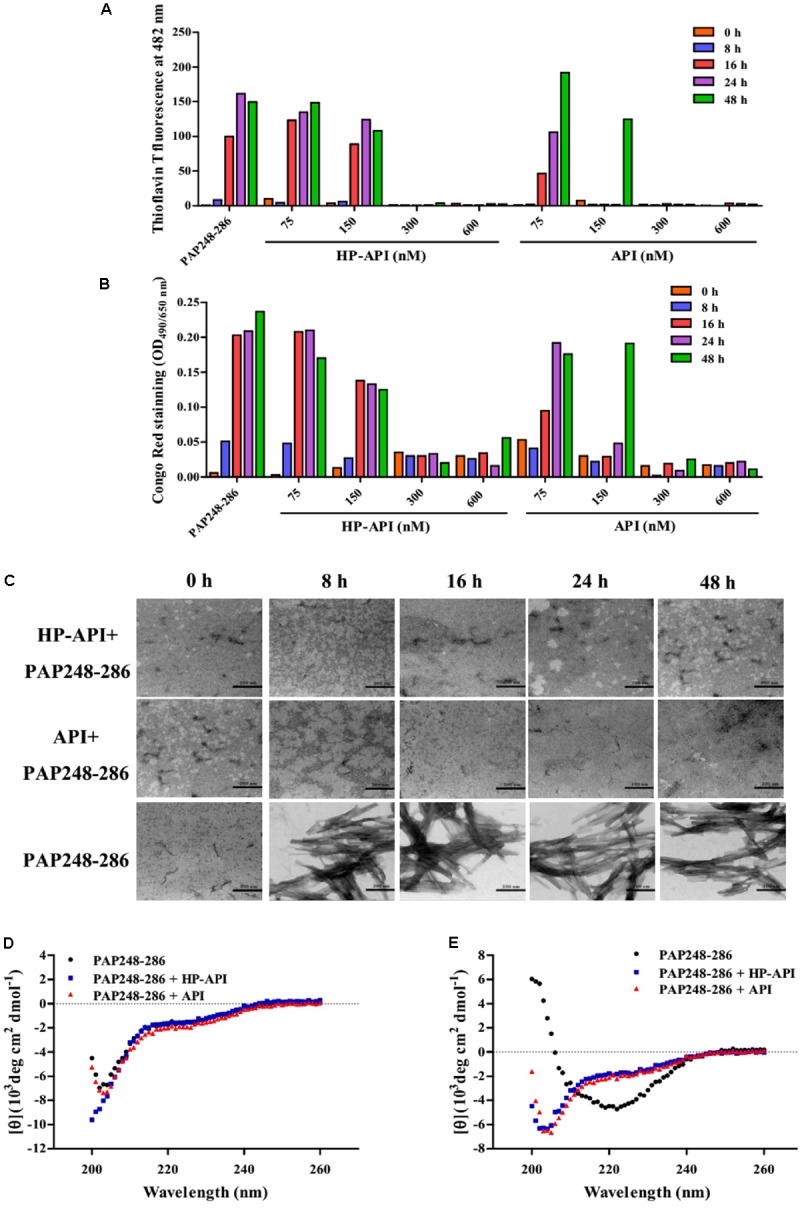
HP-API inhibited PAP248-286 from forming amyloid fibrils *in vitro*. PAP248-286-mediated formation of amyloid fibrils in the presence of HP-API was monitored by both ThT fluorescence assay **(A)** and Congo red assay **(B)**. Readings from the blank control were subtracted from all samples. **(C)** The effects of HP-API on the formation of SEVI fibrils were visualized by negative-stain TEM. The scale bar is 200 nm. HP-API inhibited beta-sheet formation of PAP 248-286 as shown by CD spectroscopy. 3 mg/ml PAP248-286 was agitated in the presence or absence of 300 nM HP-API. The spectra of samples were observed at different time points following agitation: **(D)** 0 h and **(E)** 48 h. Readings from the blank control were subtracted from all samples.

The effects of HP-API on the kinetics of SEVI fibril formation formed by peptide PAP248-286 were further detected by ThT and Congo red assay. Both HP-API and API interfered with fibrillogenesis of PAP248-286 in a dose-dependent manner, as assessed by ThT (**Figure 2A**) and Congo red assay (**Figure 2B**). At the high concentrations (600 and 300 nM), HP-API and API could completely abrogate the formation of SEVI fibrils *in vitro* even at 48 h after agitation, while PAP248-286 alone showed typical fibril growth with a lag time of approximately 8–16 h after shaking. Those results were further observed by TEM directly (**Figure 2C**). At 8 h after agitation, branching-needle-like long fibrils were revealed in the solution of PAP248-286 alone (3 mg/ml), suggesting that PAP248-286 slowly forms SEVI fibrils under agitation. However, no clear amyloid fibrils were imaged in the solution of PAP248-286 in the presence of HP-API or API (300 nM) even after shaking 48 h. All above results suggested that both HP-API and API could antagonize SEVI fibrils formation obviously.

The high-level association β-sheet secondary structure in proteins has been confirmed to be a distinguishing feature in the formation of the protein aggregates and amyloid fibrils in many diseases (Nabers et al., 2016). Here, we further detected the conformational changes associated with the aggregation of PAP248-286 in the presence or absence of HP-API shown by CD spectroscopy distinctive of random coils. As illustrated in **Figure 2D**, the spectra of PAP248-286 with or without HP-API or API at 0 h after agitation exposed a characteristic random coiled structure. After shaking at 37°C for 48 h, the spectrum of PAP248-286 alone revealed a typical β-sheet component, containing a single minimum at approximately 218 nm (**Figure 2E**). Of note, the spectra of PAP248-286 in the presence of HP-API or API at 48 h after agitation did not manifest any stable conformation, indicating that β-sheet aggregation had not occurred, which demonstrated that the addition of HP-API or API failed to induce the structural transition of PAP248-286 from random coil to a cross-β-sheet structure (**Figure 2E**).

### HP-API Neutralized SEVI-Mediated Enhancing Activity by Inhibiting the Formation of Amyloid Fibrils

**FIGURE 3:**
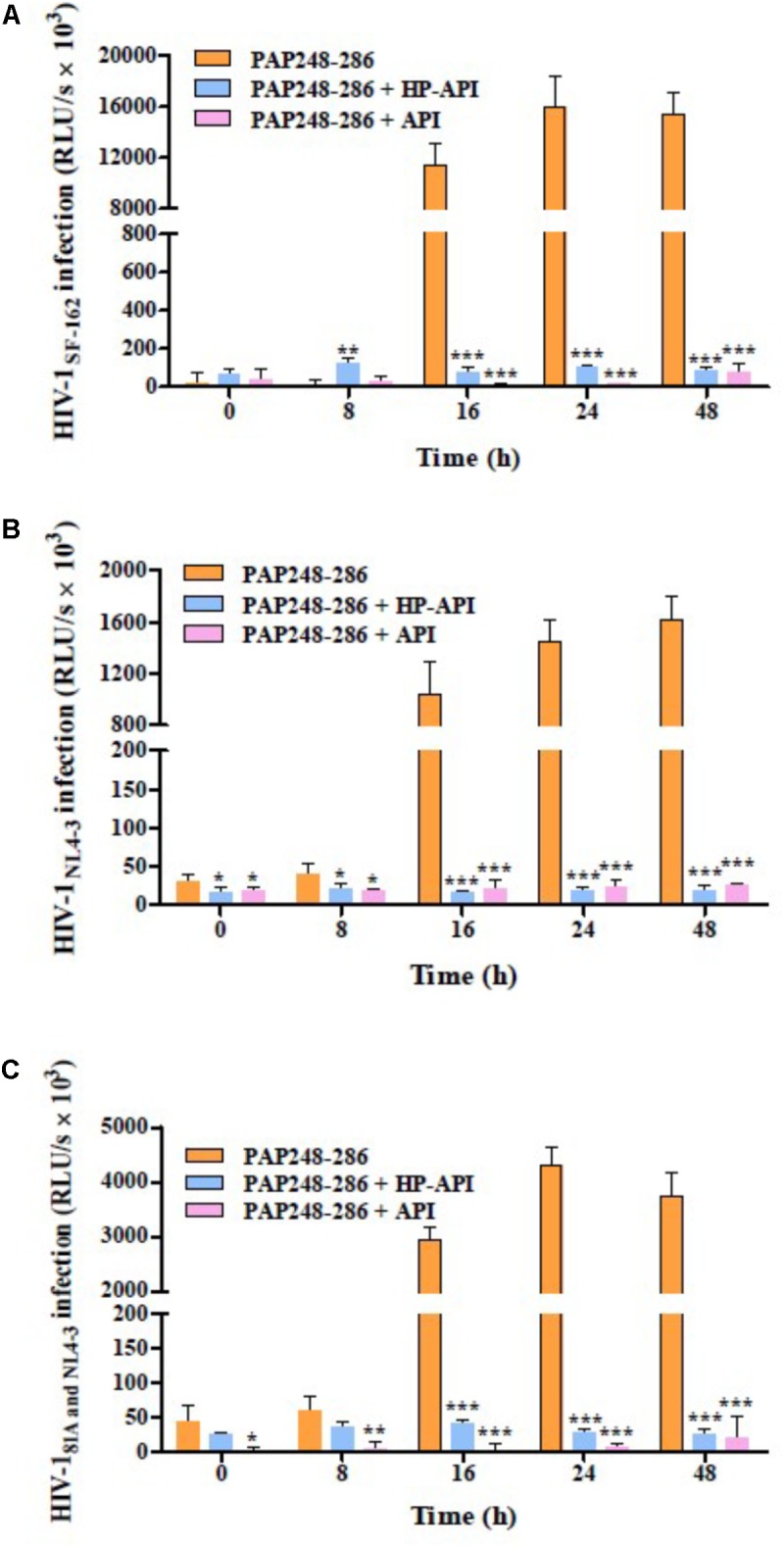
HP-API inhibited the enhancing activities of SEVI fibrils on different infectious HIV-1 clones by blocking amyloid fibrils formation. At the indicated time points, HP-API inhibited the infectivity of different infectious HIV clones, including **(A)** CCR5-tropic HIV-1_SF-162_, **(B)** CXCR4-tropic HIV-1_NL4-3_, and **(C)** Dual-tropic HIV-1_81A and NL4-3_. Data were presented in means ± SD. RLU/s, relative light units/second. A one-way ANOVA test in GraphPad Prism 5.0 (San Diego, CA, United States) was used to statistically analyze the differences between the group of SEVI fibrils with HP-API and the group of SEVI fibrils without HP-API (*^∗^p < 0.05, ^∗∗^p < 0.01, ^∗∗∗^p < 0.001*). API was used as a control.

We next focused on the effects of HP-API on SEVI-mediated enhancing activities of different infectious HIV-1 clones using a viral infection assay. For avoiding the effects of free HP-API on viral infectivity, all collected samples at different agitated time points (0, 8, 16, 24, and 48 h) as described above were centrifuged and the supernatants were discarded. The samples of PAP248-286 alone collected after shaking from 16 to 48 h could increase the infectivity of three infectious HIV-1 clones significantly, including CCR5-tropic HIV-1_SF-162_ (**Figure 3A**), CXCR4-tropic HIV-1_NL4-3_ (**Figure 3B**) and dual-tropic HIV-1_81A and NL4-3_ (**Figure 3C**). The enhancing temporal trends by a viral infection assay are coincided with the formation of SEVI amyloid fibrils by ThT, Congo red, and TEM assay (**Figure 2**). In contrast, the samples of PAP248-286 with HP-API or API (300 nM) were not contributed to the HIV-1 infection enhancement of three infectious HIV-1 clones even at agitated 48 h after agitation (**Figures 3A–C**). Those results further certified that HP-API could induce the loss of SEVI-mediated HIV-1 infection enhancing activity due to inhibiting amyloid fibrils formation by PAP248-286.

### HP-API Potently Blocked the HIV-1 Infection Enhancing Activity of Preformed SEVI Fibrils

**FIGURE 4:**
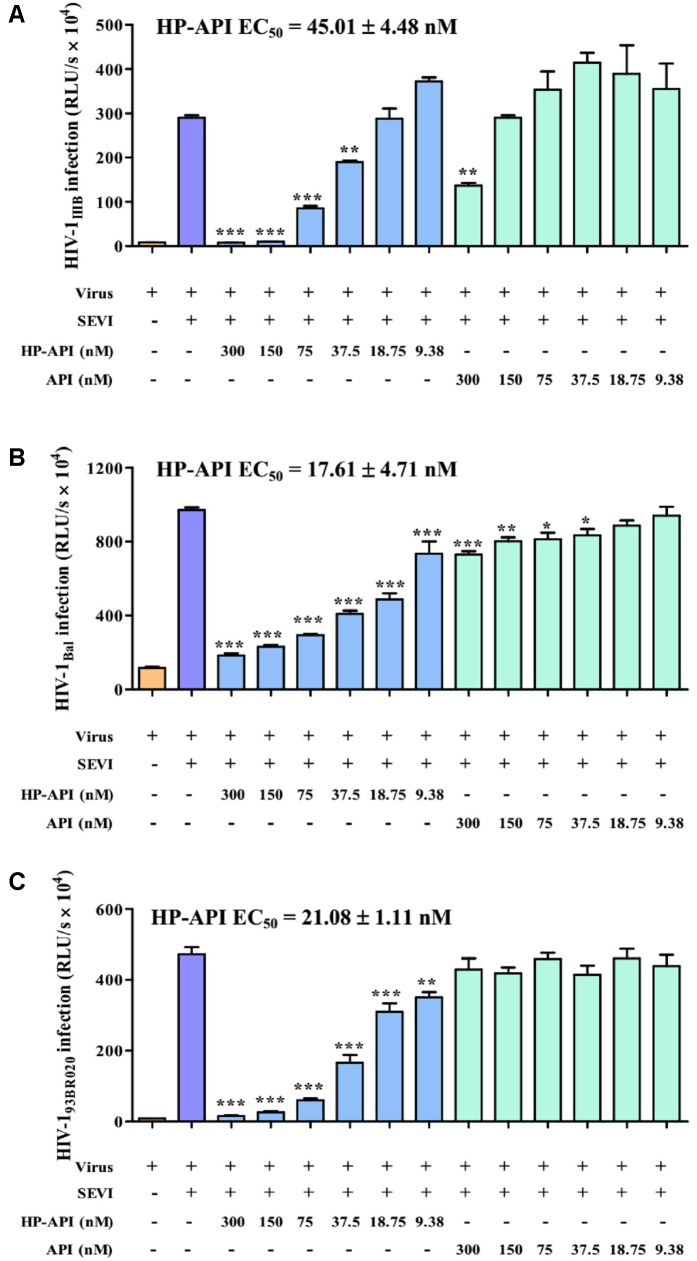
HP-API potently blocked the HIV-1 infection enhancing activity of preformed SEVI fibrils. TZM-bl cells infected by **(A)** HIV-1_IIIB_, **(B)** HIV-1_Bal_ and **(C)** HIV-1_93BR020_ in the presence of SEVI (50 μg/ml) and HP-API at various concentrations. At 3 h post-infection, the culture supernatants were changed for fresh medium. The luciferase activities of the cultures were measured at 72 h post-infection. Average values (±SD) were calculated from triplicate measurements; the data shown here represent one representative trial of three independent experiments. A one-way ANOVA test in GraphPad Prism 5.0 (San Diego, CA, United States) was used to statistically analyze the differences between samples containing SEVI alone and samples containing SEVI and HP-API (*^∗^p < 0.05, ^∗∗^p < 0.01, ^∗∗∗^p < 0.001*).

All above results demonstrated that HP-API could abrogate the formation of SEVI amyloid fibrils and block SEVI-mediated HIV infection enhancing activity because of the special antigen-antibody interaction. Here, we further detected the inhibitory activities of HP-API against infection by laboratory-adapted HIV-1 strains and primary HIV-1 isolates in the presence of preformed 50 μg/ml SEVI fibrils using a viral infection assay. As shown in **Figure 4**, SEVI fibrils could obviously enhance HIV-1 infection by different laboratory-adapted and primary strains up to 54.60 times. HP-API showed strongly inhibitory activity against infection by all tested strains, including HIV-1_IIIB_ (**Figure 4A**), HIV-1_Bal_ (**Figure 4B**), and HIV-1_93BR020_ strains (**Figure 4C**) in the presence preformed SEVI fibrils. The effective concentration for 50% inhibition (EC_50_) values of HP-API with preformed SEVI fibrils were 17.61–45.01 nM. Even though unmodified API could slightly decrease the enhanced times of HIV-1 infection by SEVI fibrils, it is regrettable that API did not exhibited anti-HIV-1 activities in the presence of SEVI fibrils even at the concentration of 300 nM (**Figure 4**).

### HP-API Curbed the Assembly and Attachment of HIV-1 Virions to SEVI Fibrils

**FIGURE 5:**
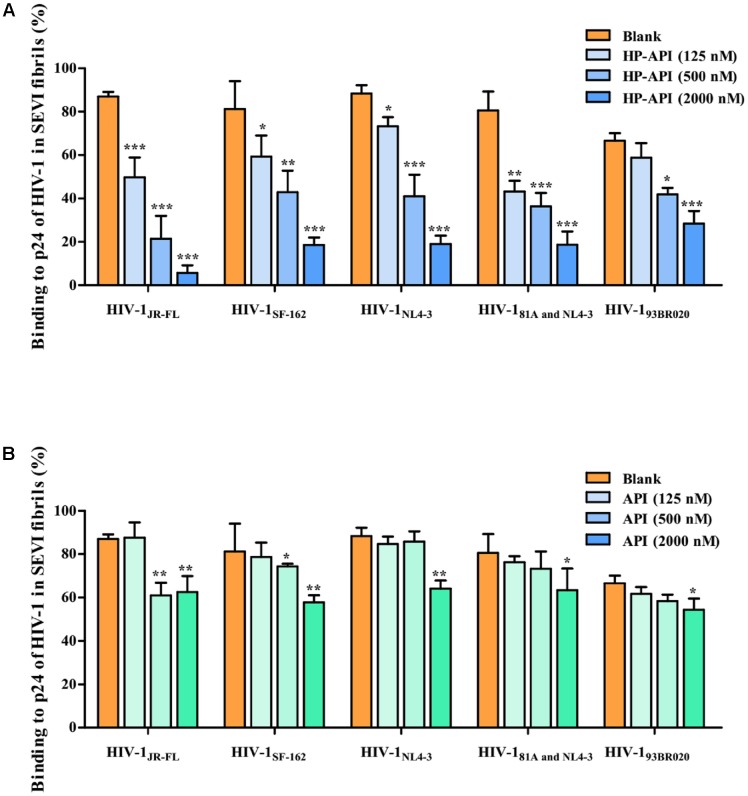
HP-API blocks the interaction of SEVI fibrils with HIV-1 virions. SEVI fibrils (200 μg/ml) were first incubated with graded concentrations of **(A)** HP-API or **(B)** API for 30 min. The samples were then centrifuged at 12,000 rpm for 5 min; the supernatant was removed, and the pellets were resuspended and mixed with HIV-1_JR-FL_, HIV-1_SF-162_, HIV-1_NL4-3_, HIV-1_81A and NL4-3_ and HIV-1_93BR020_. These mixtures were centrifuged, and the p24 antigens present in the pellet were evaluated using a p24-antigen ELISA. Average values (±SD) were calculated from triplicate measurements; the data shown here represent one representative trial of three independent experiments. A one-way ANOVA test in GraphPad Prism 5.0 (San Diego, CA, United States) was used to statistically analyze the differences between samples containing SEVI alone and samples containing SEVI and HP-API or API (*^∗^p < 0.05, ^∗∗^p < 0.01, ^∗∗∗^p < 0.001*).

Since the infectivity-enhancing activity of SEVI fibrils is associated with the electrostatic attraction between HIV-1 particles and SEVI fibrils (Roan et al., 2009, 2011), we observed the effects of HP-API and API on the binding of HIV-1 virions to SEVI fibrils by a virus pull-down assay. As demonstrated in **Figures 5A,B**, SEVI fibrils alone bound to more than 90% of the input HIV-1 virions including HIV-1 pseudoviruses, HIV-1 infectious clones and primary HIV-1 strain. It is gratifying that HP-API could significantly inhibit the binding of all of the HIV-1 virions to SEVI fibrils in a dose-dependent manner (**Figure 5A**). However, only the highest concentration of API showed slight inhibition on the binding of HIV-1 virions to SEVI fibrils (**Figure 5B**). Above results indicated that HP-API abrogated SEVI-mediated enhancement of viral infection by preventing the formation of virion-amyloid complexes.

### HP-API Showed Highly Potent Anti-HIV-1 Activities

In addition to abrogating the ability of SEVI amyloid fibrils and weakening HIV infection indirectly, HP-API also displayed a strong antiviral activity directly *in vitro*. We first evaluated the anti-HIV-1 activities of HP-API on infection by different laboratory-adapted and primary HIV-1 isolates. The unmodified API was used as a negative control, an HIV entry/fusion inhibitor T20 was chosen as a positive control. As shown in **Table 1**, HP-API showed extremely high inhibitory activities on all tested primary HIV-1 isolates with EC_50_ values in the low nM range. For the representative primary HIV-1 isolates of clades A to F and group O (X4, R5, or X4R5), the EC_50_ values of HP-API were ranged from 1.48 to 72.96 nM. However, the control unmodified API had no obviously inhibitory activity against any of the above viruses even at the highest tested concentration (3,000 nM). The anti-HIV-1 activities of the positive control T20 were also showed in **Table 1**. On the other hand, HP-API-mediated anti-HIV-1 activity was not significantly difference regardless of being pre-incubated either target cells or virus particles. HP-API was pre-incubated cells prior to infection with an EC_50_ at 2.34 ± 0.41 nM (Supplementary Figure 1B), which was similar to being pre-incubated with virus particles with an EC_50_ at 1.48 ± 0.39 nM (Supplementary Figure 1A).

**Table 1:** Inhibitory activities of HP-API on infection by laboratory-adapted and primary HIV-1 strains.

Virus strain	Inhibitory activity (Mean ± SD)^a^		
	
	HP-API	API	T20
	
	EC_50_ (nM)	EC_50_ (nM)	EC_50_ (nM)
***Laboratory-adapted HIV-1 strains***			
IIIB (clade B, X4)	1.48 ± 0.39	>3000	10.48 ± 1.61
Bal (clade B, R5)	60.99 ± 15.42	>3000	63.60 ± 1.01
***Primary HIV-1 isolates***			
92UG029 (clade A, X4)	7.16 ± 0.28	>3000	1.57 ± 0.02
33931N (clade B, R5)	6.30 ± 1.31	>3000	1.27 ± 0.19
93IN101 (clade C, R5)	21.89 ± 4.83	>3000	0.56 ± 0.01
92UG024 (clade D, X4)	36.46 ± 10.11	>3000	4.13 ± 2.92
92TH009 (clade A/E, R5)	55.99 ± 22.49	>3000	1.05 ± 0.02
NP1525 (clade A/E, X4R5)	22.34 ± 9.01	>3000	4.91 ± 3.36
93BR020 (clade F, X4R5)	72.96 ± 4.31	>3000	1.63 ± 0.81
BCF02 (clade O, R5)	40.28 ± 9.98	>3000	4.15 ± 0.21

*^a^Data are presented as means ± SD.*

### HP-API Inhibited Different Drug-Resistant HIV-1 Infection

We further detected the inhibition of HP-API on infection by the A17 strain, a non-nucleoside reverse transcriptase inhibitor (NNRTI) resistant strain, and by HIV entry/fusion inhibitor T20-resistant strains. Results showed that the drug-resistant HIV-1 strains were highly resistant to such drugs as UC781 (a NNRTI) and T20, as expected; therefore, the EC_50_ values could not be determined, even at concentrations up to 10 μM or 2 μM, respectively (**Table 2**). Notably, HP-API exhibited similar antiviral activity on wild-type HIV-1 strains and two types of drug-resistant HIV-1 strains, with EC_50_ values at the same approximate nM range.

**Table 2:** Inhibitory activity of HP-API on infection by drug-resistant HIV-1 strains.

Drug-resistant HIV-1 strains	Inhibitory activity (Mean ± SD)		
	
	HP-API	API	UC781
	
	EC_50_ (nM)	EC_50_ (nM)	EC_50_ (μM)
*A17-resistant strain*	20.91 ± 0.51	>3000	>10.00
*T20 resistant strains*	HP-API	API	T20
	EC_50_ (nM)	EC_50_ (nM)	EC_50_ (nM)
NL4-3_D36G_^a^	56.24 ± 1.32	>3000	61.66 ± 32.23
NL4-3_(36G)V 38A/N42D_	73.83 ± 2.16	>3000	>2000
NL4-3_(36G)V 38A_	79.78 ± 3.26	>3000	>2000
NL4-3_(36G)N42T/N43K_	64.96 ± 7.03	>3000	>2000
NL4-3_(36G)V 38E/N42S_	75.36 ± 0.71	>3000	>2000
NL4-3_(36G)V 38A/N42T_	67.85 ± 13.79	>3000	>2000

*^a^NL4-3_D36G_ is a T20-sensitive strain, which is the parent strain used for the generation of T20-resistant mutants.*

### HP-API Had no Cytotoxicity *in Vitro* on HIV Target Cells and Vaginal Epithelial Cells

The cytotoxicity of HP-API on target cells (MT-2, U87-CD4-CXCR4, and U87-CD4-CCR5 cells) and vaginal epithelial cells (VK2/E6E7) was detected by MTT assay. In **Table 3**, both HP-API and API at concentrations up to 3 μM had no cytotoxicity against any tested cells, which was more than 700 times its EC_90_ against HIV-1_Bal_ infection. The low cytotoxicity indicated that HP-API might be a safe microbicide when administered to the human vagina or anus.

**Table 3:** *In vitro* cytotoxicity of HP-API and API.

Cells	HP-API	API
	
	CC_50_ (nM)	CC_50_ (nM)
MT-2	>3000	>3000
U87-CD4-CXCR4	>3000	>3000
U87-CD4-CCR5	>3000	>3000
VK2/E6E7	>3000	>3000

### HP-API Inhibited HIV-1 Entry by Time-of-Addition Assay

**FIGURE 6:**
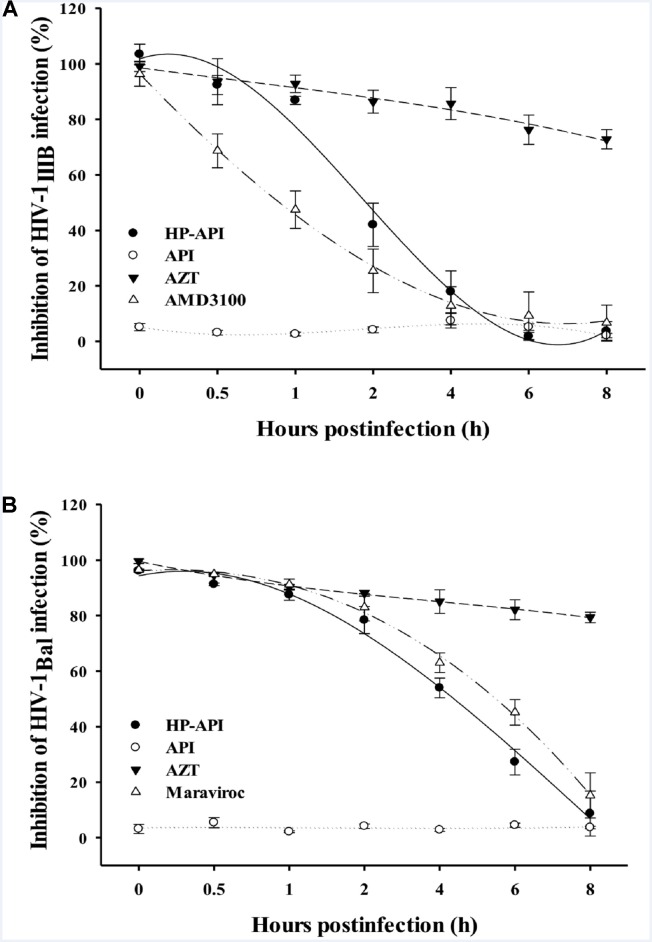
Inhibition of HIV-1 entry by HP-API as determined by time-of-addition assay. **(A)** HP-API (60 nM) or API (600 nM) was added to MT-2 cells at different intervals after infection with HIV-1_IIIB_. AZT (200 nM) and AMD3100 (100 nM) were included as controls. **(B)** HP-API (600 nM) or API (600 nM) was added to TZM-bl cells at different intervals after infection with HIV-1_Bal_. AZT (250 nM) and Maraviroc (50 nM) were included as controls. Average values (±SD) were calculated from triplicate measurements; the data shown here represent one representative trial of three independent experiments.

Our previous studies have reported that some anhydride modified proteins might be HIV entry/fusion inhibitors by blocking viral entry (Li et al., 2010; Li L. et al., 2013; Li M. et al., 2013). In order to illuminate the role of HP-API on the stages of HIV-1 infection, we first carried out a time-of-addition assay for evaluating the antiviral activities when HP-API was added to cells at different intervals post-infection. As shown in **Figures 6A,B**, a fixed concentration of the nucleoside reverse transcriptase inhibitor, AZT, showed similar antiviral activity against both HIV-1_IIIB_ and HIV-1_Bal_ when it was added to the target cells before and after viral infection. However, a fixed concentration of HIV entry inhibitors, including AMD-3100 (against HIV-1_IIIB_) and Maraviroc (against HIV-1_Bal_), exhibited significantly decreased inhibitory activity when they were added into cells at 0.5 to 2 h post-infection. Of note, HP-API showed inhibitory profiles similar to those of HIV entry inhibitors (**Figures 6A,B**), suggesting that HP-API only exerts its antiviral activities at the early stage of HIV-1 infection.

### HP-API Inhibited HIV-1 Entry by Blocking Virus–Cell Membrane Fusion

**FIGURE 7:**
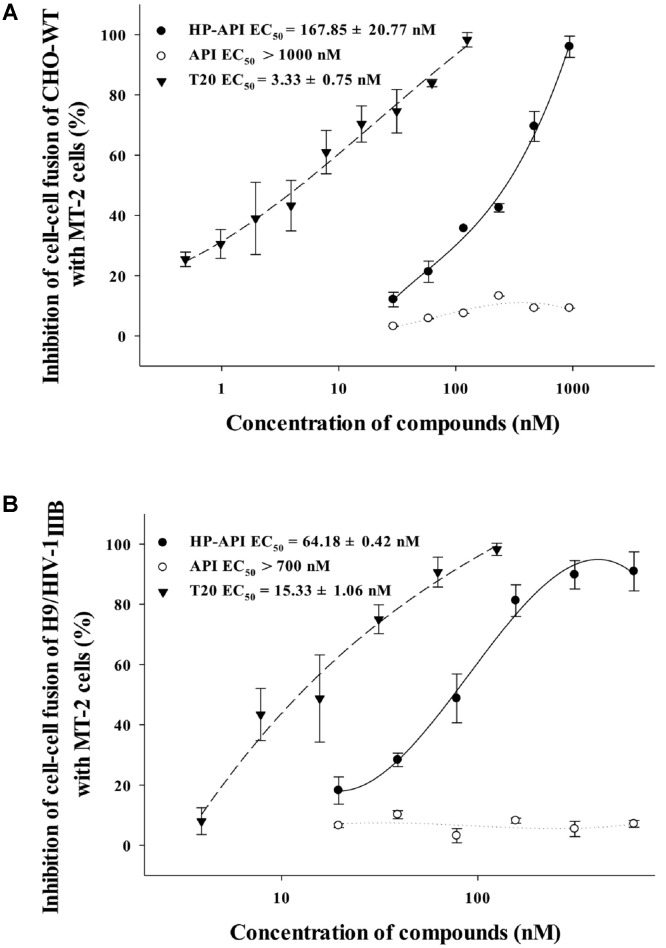
Inhibitory activity of HP-API against HIV-1-mediated cell–cell fusion. Inhibition of fusion between **(A)** CHO-WT cells or **(B)** Calcein-AM labeled HIV-1_IIIB_ infected H9 (H9/HIV-1_IIIB_) cells and MT-2 cells was assessed by a dye transfer assay as described in Section “Materials and Methods.” Average values (±SD) were calculated from triplicate measurements; the data shown here represent one representative trial of three independent experiments.

HIV Env-mediated membrane fusion is an early and a critical step of HIV entry into a target cell. We further tested the inhibition of HP-API on HIV-1 Env-mediated cell–cell fusion. Here, we chose non-infectious CHO-WT cells or infectious H9/HIV-1_IIIB_ cells as the effector cells and MT-2 cells as the target cells for simulating the early steps of the HIV-1 replication cycle. The numbers of syncytia or fluorescence-labeled fused cells were counted under an inverted microscope. As shown in **Figures 7A,B**, HP-API inhibited both the syncytium formation and the dye transfer between non-infectious or infectious effector cells and the target MT-2 cells in a dose-dependent manner. The EC_50_ values of HP-API were 167.85 nM within a non-infectious system and 64.18 nM within an infectious system, respectively. The positive control drug, T20, inhibited cell–cell fusion, with EC_50_ about 3.33 and 15.33 nM, respectively. These results indicated that HP-API might inhibit viral entry by blocking HIV-1 Env-mediated cell–cell fusion.

**FIGURE 8:**
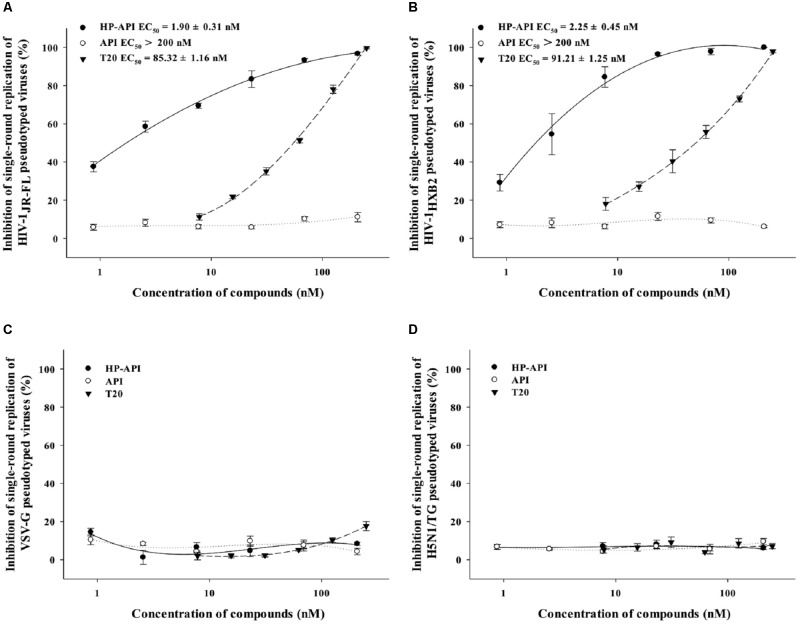
Inhibition of HP-API on single-round infection of pseudotyped viruses. **(A)** HIV-1_JR-FL_, **(B)** HIV-1_HXB2_, **(C)** VSV-G, and **(D)** H5N1/TG pseudotyped viruses were pre-incubated with graded concentrations of HP-API, API or T20 for 30 min at 37°C. Next, the mixtures were transferred to the wells with the target cells. At 72 h post-infection, luciferase activity was detected as described in Section “Materials and Methods.” Average values (±SD) were calculated from triplicate measurements; the data shown here represent one representative trial of three independent experiments.

A single-round entry assay is another commonly used method to identify a HIV entry/fusion inhibitor by assessing the effects on HIV-1 virus–cell fusion directly. Therefore, we further tested the inhibitory activity of HP-API on virus–cell fusion using pseudotyped viruses expressing HIV-1_JR-FL_ (R5 strain) and HIV-1_HXB2_ (X4 strain) Env. For analyzing the specificity of HP-API on HIV-1 Env, VSV-G pseudovirus expressing VSV Env G-protein and H5N1/TG pseudovirus expressing NA and HA from influenza were used as negative controls. T20 was used as a positive control drug. Similar to cell–cell fusion, HP-API inhibited the infection by both pseudotyped HIV-1_JR-FL_ (**Figure 8A**) and HIV-1_HXB2_ (**Figure 8B**) viruses significantly, with the EC_50_ values were 1.90 nM and 2.25 nM, respectively. Here, we further verified that HP-API could not block a single round entry of both VSV-G pseudovirus and H5N1/TG pseudovirus even at the concentration up to 200 nM (**Figures 8C,D**), indicating the specific role of HP-API in HIV-1 membrane fusion.

### HP-API Blocked gp120-CD4 Interaction by Binding Both gp120 and CD4 Molecules

**FIGURE 9:**
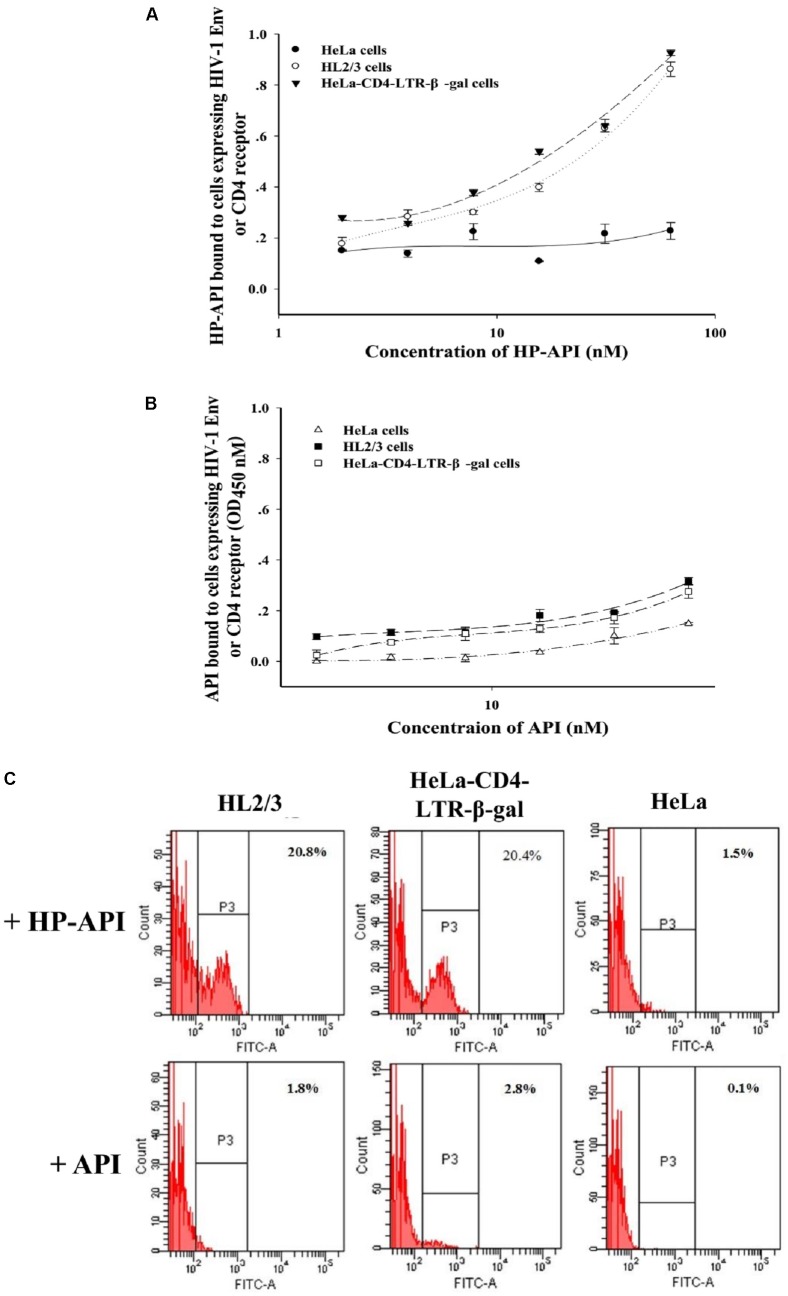
The binding of HP-API to HIV-1 Env or CD4 receptor. ELISA analysis of **(A)** HP-API or **(B)** API binding to cells expressing HIV-1 Env or CD4 molecule. HeLa-CD4-LTR-β-gal cells that express CD4 receptor were used to analyze the binding of HP-API to CD4 receptor. HL2/3 cells expressing high levels of HIV-1 Env proteins were used to evaluate the binding of HP-API to HIV-1 Env. HeLa cells were chosen as a negative control. Data were presented in means ± SD. (**C**) Flow cytometric analysis of HP-API or API binding to cells expressing HIV-1 Env or CD4 molecule. HeLa cells were chosen as a negative control.

The binding of HIV-1 Env surface subunit gp120 to CD4 receptor on a CD4^+^ target cell is the first step leading to HIV-1 viral entry. To further elucidate the binding target(s) of HP-API, the interaction of HP-API with gp120 or CD4 was examined using ELISA and flow cytometry assay. HL2/3 cells expressing high levels of HIV-1 Env proteins and HeLa-CD4-LTR-β-gal cells expressing CD4 receptor were used here. HeLa cells expressing neither HIV-1 Env nor CD4 receptor were used as a negative control. The dose-dependent binding effects of HP-API on both HL2/3 and HeLa-CD4-LTR-β-gal cells were displayed by ELISA (**Figure 9A**), suggesting its binding to both HIV-1 Env and CD4 receptor. However, the negative control, API, could not bind to above cells (**Figure 9B**). Similar results showed by flow cytometry assay. HP-API could significantly bind to both HL2/3 and HeLa-CD4-LTR-β-gal cells, while it had only background binding to HeLa cells (**Figure 9C**).

**FIGURE 10:**
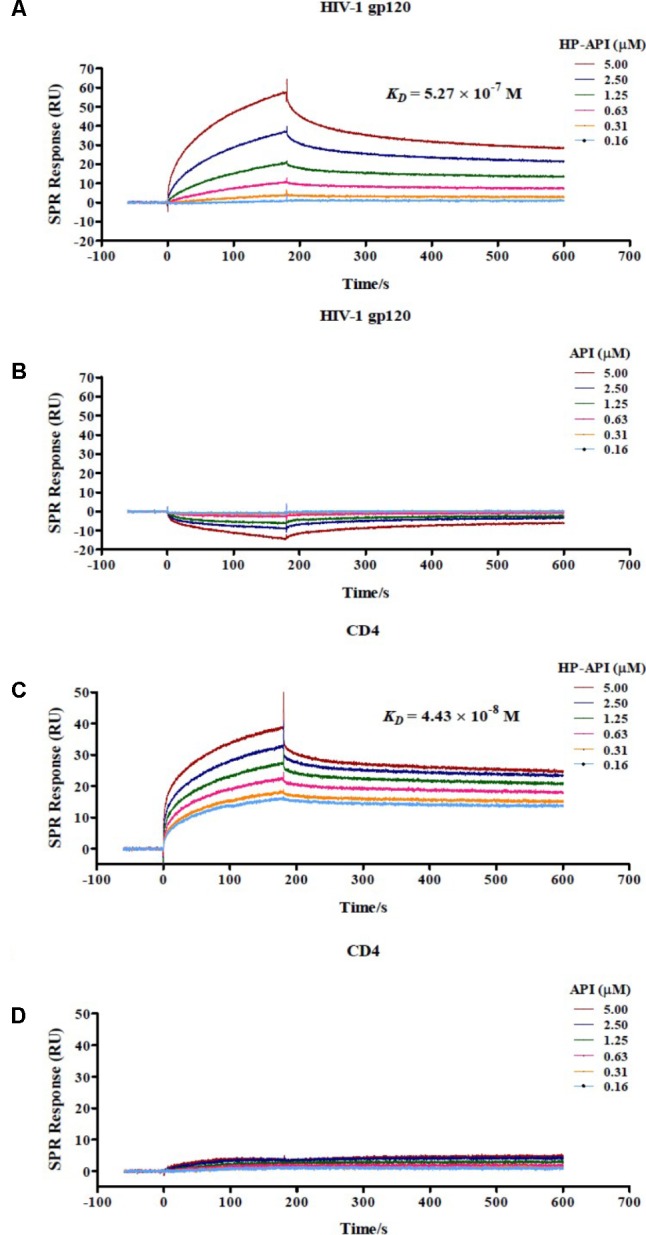
Binding of HP-API to HIV-1 gp120 and CD4 as assessed by SPR assay. The binding of **(A)** HP-API or **(B)** API to HIV-1 gp120 and the binding of **(C)** HP-API or **(D)** API to CD4. HIV-1 gp120 (20 μg/ml) and CD4 (6 μg/ml) were immobilized on a sensor chip. Subsequently, twofold serial dilutions of HP-API or API were injected as the analyte. The affinity constant *K_D_* is the ratio of the dissociation constant *K_d_* to the association constant *K_a_* (*K_D_* = *K_d_*/*K_a_*). The response units were recorded against the flow time (s).

For further determining if HP-API indeed binds specifically to both CD4 and gp120, the interaction of HP-API with gp120 from the HIV-1_JR-FL_ or recombinant human CD4 was examined using a SPR assay. As shown in **Figures 10A,C**, HP-API could bind to both gp120 and CD4 with a binding affinity (*K*_D_) of 5.27 × 10^-7^ M and 4.43 × 10^-8^ M, respectively. However, API could neither bind to gp120 nor CD4 even at the concentration of 5 μM (**Figures 10B,D**). These results confirmed that HP-API could bind to both gp120 and CD4 specifically and then interfere with their interaction, resulting in the inhibition of HIV-1 entry.

## Discussion

Although combination antiretroviral therapy (cART) is widely used in AIDS patients, it is limited by the expensive cost, side effects, viral mutation or drug resistance (McArthur et al., 2003; Nasir et al., 2017). HIV-1 entry/fusion inhibitors have the potential to block the earliest step of viral infection and intercept the virus before it invades target cells (Xu et al., 2017). Microbicides are a class of HIV entry inhibitor that can be controlled by women and prevent HIV infection in the vagina or rectum before sexual intercourse (Garg et al., 2009). Unfortunately, after two decades of researches on topical microbicides, only limited success has been achieved, calling for further efforts to find effective microbicides (Garg et al., 2009; Buckheit et al., 2010; Vanpouille et al., 2012; McGowan, 2014). Continual failures of those microbicide candidates in clinical trials implied that they share some underlying mechanism that impairs efficacy in their application to human beings. Identification of some endogenous components in the host environment that affect the efficacy of microbicides is critical in the spread of HIV/AIDS (Tan et al., 2013; Zirafi et al., 2014). Notably, SEVI amyloid fibrils formed by PAP248-286 in human semen might be one of major causes to clinical trial failures of microbicide candidates during sexual intercourse (Munch et al., 2007; Kim et al., 2010).

Several studies have demonstrated that SEVI amyloid fibrils in semen could capture HIV-1 virions leading to localized enrichment of the surface of amyloid fibrils and promote HIV-1 infection efficiently (Munch et al., 2007; Roan et al., 2009). The strategy of neutralizing the enhancing activity of SEVI fibrils might be an attractive option for the development of new microbicides in future. Some compounds might be ideal SEVI antagonist-based microbicides by blocking the formation of SEVI or disassembling pre-formed SEVI fibrils. A natural ingredient of green tea, epigallocatechin-3-gallate (EGCG), seems to be an ideal antiretroviral agent by abrogating human and macaque SEVI-mediated enhancement of HIV-1 infection (Popovych et al., 2012; Castellano et al., 2015b; Zhou et al., 2017). An amyloid-remodeling nanomachine from yeast, Hsp104, antagonized semen-derived amyloid fibrils and reduced HIV transmission (Castellano et al., 2015a). A molecular tweezer, CLR01, disrupted the formation of SEVI fibrils and remodeled pre-formed SEVI fibrils (Lump et al., 2015). The generation and characterization of conformation-specific antibodies for specifically recognizing SEVI fibrils may also be a good choice. A promising strategy is to explore a bifunctional microbicide possessing both antiviral and anti-SEVI activities.

Therefore, we hypothesized that a specific anti-PAP antibody might interfere with SEVI-mediated enhancement of HIV activity by binding to PAP248-286 and SEVI fibrils. Preliminary results showed that an anti-PAP IgG (API) did bind to both PAP248-286 and SEVI fibrils, while it showed no anti-HIV activity against any tested HIV-1 isolates, even at concentration up to 3000 nM (**Table 1**). Of note, our previous studies have reported that an inactive protein could be converted into an effective antiviral compound dramatically by anhydride modification on the site-specific amino acids (Li et al., 2010; Li L. et al., 2013; Li M. et al., 2013). Based on this idea, we prepared HP-API for obtaining a new bifunctional microbicide with both anti-SEVI and anti-HIV activities. Interestingly, HP-API could significantly inhibit infection by the representative laboratory-adapted and primary HIV-1 isolates in the presence of SEVI fibrils (**Figure 4**). Those results suggest that HP-API might be worthy of further exploration for developing an ideal bifunctional microbicide candidate.

Here, we first demonstrated that HP-API could retain specific antigen-antibody binding activity after anhydride modification. ELISA and ITC assay results showed that the antigen-antibody interaction was not affected by anhydride modification (**Figure 1**). Aggregation kinetics of SEVI fibrils showed that HP-API at high concentrations could completely abrogate the formation of SEVI fibrils *in vitro*, even at 48 h after agitation, while PAP248-286 alone showed typical fibril growth with a lag time of approximately 8–16 h after shaking (**Figures 2A,B**). The inhibition of HP-API on PAP248-286 aggregation at different agitated time points was confirmed directly by TEM, no clear amyloid fibrils of PAP248-286 with HP-API were imaged even after shaking 48 h (**Figure 2C**). These evidences suggested that HP-API could antagonize the formation of SEVI fibrils. Interestingly, since HP-API inhibited the formation of SEVI fibrils, the enhancing activity of these SEVI fibrils on all tested HIV-1 infectious clones was significantly impaired (**Figure 3**). The trend of temporal enhancement of SEVI fibrils is coincident with PAP248-286 aggregates containing HP-API, as shown in **Figures 2A,B**.

In general, the aggregation of proteins always forms thread-like or straight amyloid fibrils, which are insoluble and resistant to protease activity (House et al., 2009). The high-level association β-sheet secondary structure in proteins has been confirmed to be a distinguishing feature in the formation of the protein aggregates and amyloid fibrils in many diseases (Nabers et al., 2016). A highly stable cross-β-sheet secondary structure of amyloid fibrils is usually identified by CD spectra assay (Sheik et al., 2017). However, PAP248-286 monomer lacks a significant proportion of ordered cross-β-sheet structure (**Figure 2D**). Here, HP-API inhibited the association β-sheet secondary structure in PAP248-286 after agitating together for 48 h (**Figure 2E**).

Several researches have certified that SEVI fibrils in human semen could capture HIV-1 virions, resulting in their local enrichment at the amyloid surface and, hence, efficiently promoting HIV-1 infection (Munch et al., 2007; Roan et al., 2009). Generally, numerous HIV virions might be required to establish a productive HIV infection. Surprisingly, just one to three infectious virions are adequate to HIV infection in the presence of SEVI fibrils. Previous studies have shown that SEVI fibrils possess intrinsic positive charges that ultimately facilitate virus attachment to target cells. It is gratifying that HP-API could significantly inhibit the binding of all these HIV-1 virions to SEVI fibrils in a dose-dependent manner (**Figures 4, 5A**). Unfortunately, we did not observe the obvious inhibition of unmodified API on the binding of HIV-1 virions to SEVI fibrils and the infection in the presence of performed SEVI fibrils (**Figures 4, 5B**). The most possible reason is that HP-API is a highly negatively charged compound, in which the positively charged side chains were converted to negatively charged side chains after modification by HP. The electrostatic interaction between HP-API and SEVI fibrils might cause HP-API shield the surface cationic property of SEVI fibrils, leading to competitive inhibition of the binding of SEVI to virus. Above results indicated that HP-API might act as an efficient inhibitor of SEVI formation at the early stages of PAP248-286 aggregation and then abrogate the capability of SEVI fibrils to entrap HIV-1 virions. Surprisingly, the inhibition of HIV-1 infection by HP-API was also observed in the absence of SEVI fibrils, suggesting that mechanism of HP-API inhibition was independent of its ability to prevent SEVI fibril formation.

The failure of some microbicide candidates in clinical trials was a forewarning that highly effective and broadly antiviral activity and low toxicity are necessary (Fernandez Romero et al., 2014). Therefore, we further detected the inhibition of HP-API on HIV-1 infection by distinct HIV-1 strains. As shown in **Table 1**, HP-API exhibited strong antiviral activity on all tested HIV-1 strains, with EC_50_ in the low nM range. Notably, HP-API also effectively inhibited infection by HIV-1 variants resistant to a NRTI and a HIV entry inhibitor (**Table 2**), indicating that HP-API may be used in combination with antiretroviral (ARV)-based or entry inhibitor-based microbicide candidates to achieve synergistic antiviral activity or reduce the potential toxic effects from dose reduction. The potential safety evaluation *in vitro* showed that HP-API had no cytotoxicity at tested concentration to HIV target cells or vaginal epithelial cells (**Table 3**).

Although HP-API possesses a strong antiviral property, its detailed mechanism of action is still elusive. The entry of HIV to host cells is mediated by the envelope protein(s) of HIV. The first step is the binding of envelope glycoprotein gp120 to the initial receptor/co-receptors, CD4, CCR5 or CXCR4 (Wu et al., 2017), induces the start of a cascade of conformational changes in gp120 and gp41, bringing the viral and host cell membranes to sufficient proximity and fusion (Madani et al., 2016). Our previous studies reported that a series of chemically modified proteins inhibited HIV-1 fusion/entry by targeting both HIV-1 gp120 envelope and CD4 receptor (Li et al., 2010). Like those chemically modified proteins, our experiments also confirmed that HP-API was an HIV-1 entry inhibitor. Time-of-addition assay showed that HP-API exerted its strongest antiviral activity when it was added to the system at pre-infection, followed by a significantly decreased inhibitory activity at 2 h post-infection (**Figure 6**). HIV-1-mediated cell–cell membrane fusion (**Figure 7**) and a single-round entry assay (**Figure 8**) further confirmed that HP-API might be an HIV-1 fusion/entry inhibitor. It is well known that HIV-1 entry into the target cell is mediated by HIV-1 envelope proteins and host CD4 receptor. Our cell- based ELISA (**Figures 9A,B**) and flow cytometry assays (**Figure 9C**), suggesting that HP-API could obviously bind to both HIV-1 gp120 envelope and CD4 receptor. Using a SPR assay, we demonstrated that HP-API could bind to both CD4 and HIV-1 gp120 specifically, thereby inhibiting the interaction between gp120 and CD4 (**Figure 10**).

Previous studies have reported that Arg59 residue of CD4 plays a critical role in the CD4 for conformational changes in gp120 during the sequential process of entry and infection by HIV-1 (Siddiqi et al., 1997; Fontenot et al., 2007). Furthermore, the lysine residue at position 169 (K169) in the second variable region (V2) of gp120 is crucial to HIV infection (Schwalbe and Schreiber, 2015; Wiehe and Nicely, 2017). One of the main reasons contributing to specific binding to CD4 or gp120 may be attributed to the highly negatively charged of HP-API. Some important and positively charged amino acids (lysine and arginine) in CD4 or gp120 during HIV infection might be bound by HP-API. Our group previously reported that an anhydride-modified protein (HP-OVA) had no deleterious effect on the function of CD4^+^ T cells or other host cells, especially for those circulating in the human body (Li et al., 2010). However, we cannot exclude the possibility that long-term use of HP-API may suppress the function of other host cell surface proteins with positively charged amino acids.

Recently, monoclonal antibodies have a good potential to be developed into a new class of anti-HIV drugs. Preliminary data have consistently shown that ibalizumab (formerly TNX-355), an anti-CD4 monoclonal antibody, possessed effective anti-HIV-1 activity and minor adverse effects in undergoing phase 2 and 3 clinical trials. Ibalizumab inhibits HIV entry by inducing conformational changes of the CD4 receptors and gp120 that prevent post-CD4 binding events without eliciting an immunosuppressive MHC II response (Iacob and Iacob, 2017). It is worth noting that HP-API is also an anti-CD4 antibody and might have the similar advantages to ibalizumab, such as lack of toxicity, good resistance and ability to restore CD4 T-cell responses (Jacobson et al., 2009).

## Conclusion

Taken together, the easy preparation, high antiviral activity, strong anti-SEVI-mediated HIV enhancement, and low cytotoxicity all make HP-API worth investigating for development of an ideal bifunctional microbicide candidate with both anti-HIV and anti-SEVI activity.

## Ethics Statement

The research protocols for this study were approved by the Ethical Committee of Nanfang Hospital and performed in accordance with relevant guidelines and regulations.

## Author Contributions

LL, XZ, and JC conceived the project and wrote the manuscript. XZ, JC, FY, and RR performed the experiments. CW, QW, and ST analyzed the data. All authors reviewed the manuscript. LL, SJ, and SL supervised the project and revised the manuscript.

## Conflict of Interest Statement

The authors declare that the research was conducted in the absence of any commercial or financial relationships that could be construed as a potential conflict of interest.

## References

[B1] Arnold F. Schnell J. Zirafi O. Sturzel C. Meier C. Weil T. (2012). Naturally occurring fragments from two distinct regions of the prostatic acid phosphatase form amyloidogenic enhancers of HIV infection. *J. Virol.* 86 1244–1249. 10.1128/jvi.06121-11 22090109 PMC3255800

[B2] Avramis V. I. Avramis E. V. Hunter W. Long M. C. (2009). Immunogenicity of native or pegylated *E. coli* and Erwinia asparaginases assessed by ELISA and surface plasmon resonance (SPR-biacore) assays of IgG antibodies (Ab) in sera from patients with acute lymphoblastic leukemia (ALL). *Anticancer Res.* 29 299–302. 19331165

[B3] Buckheit R. W. Jr. Watson K. M. Morrow K. M. Ham A. S. (2010). Development of topical microbicides to prevent the sexual transmission of HIV. *Antiviral Res.* 85 142–158. 10.1016/j.antiviral.2009.10.013 19874851 PMC2815091

[B4] Castellano L. M. Bart S. M. Holmes V. M. Weissman D. Shorter J. (2015a). Repurposing Hsp104 to antagonize seminal amyloid and counter HIV infection. *Chem. Biol.* 22 1074–1086. 10.1016/j.chembiol.2015.07.007 26256479 PMC4546571

[B5] Castellano L. M. Hammond R. M. Holmes V. M. Weissman D. Shorter J. (2015b). Epigallocatechin-3-gallate rapidly remodels PAP85-120, SEM1(45-107), and SEM2(49-107) seminal amyloid fibrils. *Biol. Open* 4 1206–1212. 10.1242/bio.010215 26319581 PMC4582112

[B6] Chen J. Ren R. Tan S. Zhang W. Zhang X. Yu F. (2015). A peptide derived from the HIV-1 gp120 coreceptor-binding region promotes formation of PAP248-286 amyloid fibrils to enhance HIV-1 infection. *PLoS One* 10:e0144522. 10.1371/journal.pone.0144522 26656730 PMC4687630

[B7] Doncel G. F. Joseph T. Thurman A. R. (2011). Role of semen in HIV-1 transmission: inhibitor or facilitator? *Am. J. Reprod. Immunol.* 65 292–301. 10.1111/j.1600-0897.2010.00931.x 21087339

[B8] Fernandez Romero J. A. Gil P. I. Re V. Robbiani M. Paglini G. (2014). Microbicides for preventing sexually transmitted infections: current status and strategies for preclinical evaluation of new candidates. *Rev. Argent. Microbiol.* 46 256–268. 10.1016/s0325-7541(14)70080-x 25444135

[B9] Fontenot D. Jones J. K. Hossain M. M. Nehete P. N. Vela E. M. Dwyer V. A. (2007). Critical role of Arg59 in the high-affinity gp120-binding region of CD4 for human immunodeficiency virus type 1 infection. *Virology* 363 69–78. 10.1016/j.virol.2006.12.003 17320923

[B10] French K. C. Roan N. R. Makhatadze G. I. (2014). Structural characterization of semen coagulum-derived SEM1(86-107) amyloid fibrils that enhance HIV-1 infection. *Biochemistry* 53 3267–3277. 10.1021/bi500427r 24811874 PMC4039337

[B11] Garg A. B. Nuttall J. Romano J. (2009). The future of HIV microbicides: challenges and opportunities. *Antivir. Chem. Chemother.* 19 143–150. 10.1177/095632020901900401 19374141

[B12] House E. Mold M. Collingwood J. Baldwin A. Goodwin S. Exley C. (2009). Copper abolishes the beta-sheet secondary structure of preformed amyloid fibrils of amyloid-beta(42). *J. Alzheimers Dis.* 18 811–817. 10.3233/jad-2009-1235 19749401 PMC2857508

[B13] Iacob S. A. Iacob D. G. (2017). Ibalizumab targeting CD4 receptors, an emerging molecule in HIV therapy. *Front. Microbiol.* 8 2323. 10.3389/fmicb.2017.02323 29230203 PMC5711820

[B14] Jacobson J. M. Kuritzkes D. R. Godofsky E. DeJesus E. Larson J. A. Weinheimer S. P. (2009). Safety, pharmacokinetics, and antiretroviral activity of multiple doses of ibalizumab (formerly TNX-355), an anti-CD4 monoclonal antibody, in human immunodeficiency virus type 1-infected adults. *Antimicrob. Agents Chemother.* 53 450–457. 10.1128/aac.00942-08 19015347 PMC2630626

[B15] Kim K. A. Yolamanova M. Zirafi O. Roan N. R. Staendker L. Forssmann W. G. (2010). Semen-mediated enhancement of HIV infection is donor-dependent and correlates with the levels of SEVI. *Retrovirology* 7:55. 10.1186/1742-4690-7-55 20573198 PMC2914040

[B16] Li L. He L. Tan S. Guo X. Lu H. Qi Z. (2010). 3-hydroxyphthalic anhydride-modified chicken ovalbumin exhibits potent and broad anti-HIV-1 activity: a potential microbicide for preventing sexual transmission of HIV-1. *Antimicrob. Agents Chemother.* 54 1700–1711. 10.1128/aac.01046-09 20194691 PMC2863607

[B17] Li L. Qiu J. Lu L. An S. Qiao P. Jiang S. (2013). 3-Hydroxyphthalic anhydride-modified human serum albumin as a microbicide candidate inhibits HIV infection by blocking viral entry. *J. Antimicrob. Chemother.* 68 573–576. 10.1093/jac/dks458 23221626

[B18] Li M. Duan J. Qiu J. Yu F. Che X. Jiang S. (2013). 3-hydroxyphthalic anhydride-modified human serum albumin as a microbicide candidate against HIV type 1 entry by targeting both viral envelope glycoprotein gp120 and cellular receptor CD4. *AIDS Res. Hum. Retroviruses* 29 1455–1464. 10.1089/aid.2013.0043 23711095 PMC3809378

[B19] Lump E. Castellano L. M. Meier C. Seeliger J. Erwin N. Sperlich B. (2015). A molecular tweezer antagonizes seminal amyloids and HIV infection. *Elife* 4: e05397. 10.7554/eLife.05397 26284498 PMC4536748

[B20] Madani N. Princiotto A. M. Easterhoff D. Bradley T. Luo K. Williams W. B. (2016). Antibodies elicited by multiple envelope glycoprotein immunogens in primates neutralize primary human immunodeficiency viruses (HIV-1) sensitized by CD4-mimetic compounds. *J. Virol.* 90 5031–5046. 10.1128/jvi.03211-15 26962221 PMC4859707

[B21] McArthur J. C. Haughey N. Gartner S. Conant K. Pardo C. Nath A. (2003). Human immunodeficiency virus-associated dementia: an evolving disease. *J. Neurovirol.* 9 205–221. 10.1080/13550280390194109 12707851

[B22] McGowan I. (2014). The development of rectal microbicides for HIV prevention. *Expert Opin. Drug Deliv.* 11 69–82. 10.1517/17425247.2013.860132 24266648

[B23] Munch J. Rucker E. Standker L. Adermann K. Goffinet C. Schindler M. (2007). Semen-derived amyloid fibrils drastically enhance HIV infection. *Cell* 131 1059–1071. 10.1016/j.cell.2007.10.014 18083097

[B24] Nabers A. Ollesch J. Schartner J. Kotting C. Genius J. Hafermann H. (2016). Amyloid-beta-secondary structure distribution in cerebrospinal fluid and blood measured by an immuno-infrared-sensor: a biomarker candidate for Alzheimer’s disease. *Anal. Chem.* 88 2755–2762. 10.1021/acs.analchem.5b04286 26828829

[B25] Nasir I. A. Emeribe A. U. Ojeamiren I. Aderinsayo Adekola H. (2017). Human immunodeficiency virus resistance testing technologies and their applicability in resource-limited settings of Africa. *Infect. Dis.* 10:1178633717749597. 10.1177/1178633717749597 29308013 PMC5751912

[B26] Popovych N. Brender J. R. Soong R. Vivekanandan S. Hartman K. Basrur V. (2012). Site specific interaction of the polyphenol EGCG with the SEVI amyloid precursor peptide PAP(248-286). *J. Phys. Chem. B* 116 3650–3658. 10.1021/jp2121577 22360607 PMC3310975

[B27] Roan N. R. Muller J. A. Liu H. Chu S. Arnold F. Sturzel C. M. (2011). Peptides released by physiological cleavage of semen coagulum proteins form amyloids that enhance HIV infection. *Cell Host Microbe* 10 541–550. 10.1016/j.chom.2011.10.010 22177559 PMC3257029

[B28] Roan N. R. Munch J. Arhel N. Mothes W. Neidleman J. Kobayashi A. (2009). The cationic properties of SEVI underlie its ability to enhance human immunodeficiency virus infection. *J. Virol.* 83 73–80. 10.1128/jvi.01366-08 18945786 PMC2612336

[B29] Sabatte J. Remes Lenicov F. Cabrini M. Rodriguez Rodrigues C. Ostrowski M. Ceballos A. (2011). The role of semen in sexual transmission of HIV: beyond a carrier for virus particles. *Microbes Infect.* 13 977–982. 10.1016/j.micinf.2011.06.005 21767659

[B30] Saidi H. Jenabian M. A. Belec L. (2012). Understanding factors that modulate HIV infection at the female genital tract mucosae for the rationale design of microbicides. *AIDS Res. Hum. Retroviruses* 28 1485–1497. 10.1089/aid.2012.0049 22867060

[B31] Schwalbe B. Schreiber M. (2015). Effect of lysine to arginine mutagenesis in the V3 loop of HIV-1 gp120 on viral entry efficiency and neutralization. *PLoS One* 10:e0119879. 10.1371/journal.pone.0119879 25785610 PMC4364900

[B32] Sheik D. A. Chamberlain J. M. Brooks L. Clark M. Kim Y. H. Leriche G. (2017). Hydrophobic nanoparticles reduce the beta-sheet content of SEVI amyloid fibrils and inhibit SEVI-enhanced HIV infectivity. *Langmuir* 33 2596–2602. 10.1021/acs.langmuir.6b04295 28207276

[B33] Siddiqi M. A. Tachibana M. Ohta S. Ikegami Y. Tahara-Hanaoka S. Huang Y. Y. (1997). Comparative analysis of the gp120-binding area of murine and human CD4 molecules. *J. Acquir. Immune Defic. Syndr. Hum. Retrovirol.* 14 7–12. 10.1097/00042560-199701010-00002 8989204

[B34] Tan S. Lu L. Li L. Liu J. Oksov Y. Lu H. (2013). Polyanionic candidate microbicides accelerate the formation of semen-derived amyloid fibrils to enhance HIV-1 infection. *PLoS One* 8:e59777. 10.1371/journal.pone.0059777 23544097 PMC3609764

[B35] UNAIDS (2016). *AIDS by the Numbers 2016.* Available at: http://www.unaids.org/sites/default/files/media_asset/AIDS_by_the_numbers_2016_en

[B36] Usmani S. M. Zirafi O. Muller J. A. Sandi-Monroy N. L. Yadav J. K. Meier C. (2014). Direct visualization of HIV-enhancing endogenous amyloid fibrils in human semen. *Nat. Commun.* 5:3508. 10.1038/ncomms4508 24691351 PMC4129123

[B37] Vanpouille C. Arakelyan A. Margolis L. (2012). Microbicides: still a long road to success. *Trends Microbiol.* 20 369–375. 10.1016/j.tim.2012.05.005 22705107 PMC3756685

[B38] Wiehe K. Nicely N. I. (2017). Immunodominance of antibody recognition of the HIV envelope V2 region in Ig-humanized mice. *J. Immunol.* 198 1047–1055. 10.4049/jimmunol.1601640 28011932 PMC5262538

[B39] Wu B. Ouyang Z. Lyon C. J. Zhang W. Clift T. Bone C. R. (2017). Plasma levels of complement factor I and C4b peptides are associated with HIV suppression. *ACS Infect. Dis.* 3 880–885. 10.1021/acsinfecdis.7b00042 28862830 PMC5727467

[B40] Xu F. Acosta E. P. Liang L. He Y. Yang J. Kerstner-Wood C. (2017). Current Status of the pharmacokinetics and pharmacodynamics of HIV-1 entry inhibitors and HIV therapy. *Curr. Drug Metab.* 18 769–781. 10.2174/1389200218666170724112412 28738768

[B41] Xun T. Li W. Chen J. Yu F. Xu W. Wang Q. (2015). ADS-J1 inhibits semen-derived amyloid fibril formation and blocks fibril-mediated enhancement of HIV-1 infection. *Antimicrob. Agents Chemother.* 59 5123–5134. 10.1128/aac.00385-15 26055369 PMC4538458

[B42] Zhou R. H. Guo L. Liu J. B. Liu H. Hou W. Ma T. C. (2017). Epigallocatechin gallate inhibits macaque SEVI-mediated enhancement of SIV or SHIV infection. *J. Acquir. Immune Defic. Syndr.* 75 232–240. 10.1097/qai.0000000000001361 28328549 PMC5429200

[B43] Zirafi O. Kim K. A. Roan N. R. Kluge S. F. Muller J. A. Jiang S. (2014). Semen enhances HIV infectivity and impairs the antiviral efficacy of microbicides. *Sci. Transl. Med.* 6:262ra157. 10.1126/scitranslmed.3009634 25391483 PMC4372245

