# Supplementary material for: 3-Hydroxyphthalic Anhydride- Modified Rabbit Anti-PAP IgG as a Potential Bifunctional HIV-1 Entry Inhibitor
Source: Front Microbiol. 2018 Jun 19;9:1330. doi: 10.3389/fmicb.2018.01330 (PMC6018217; doi:10.3389/fmicb.2018.01330)
Supplement: Supplementary file 1 [file Presentation_1.PDF]

Supplementary information

3-Hydroxyphthalic anhydride-modified rabbit anti-PAP IgG as a potential bifunctional HIV-1 entry inhibitor

Xuanxuan Zhang [a, †], ***Jinquan Chen*** [a, b, †], ***Fei Yu*** [c, d, †], ***Chunyan Wang*** [e], ***Ruxia Ren*** [a], ***Qian Wang*** [c], ***Suiyi Tan*** [a], ***Shibo Jiang*** [c, f], ***Shuwen Liu*** [a,*], ***Lin Li*** [a,*]

[a] *Guangdong Provincial Key Laboratory of New Drug Screening, Guangzhou Key Laboratory of Drug Research for Emerging Virus Prevention and Treatment, School of Pharmaceutical Sciences, Southern Medical University, Guangzhou, 510515, China;*

[b] *Jiangsu Food & Pharmaceutical Science College, Huai'an, 223003, China;*

[c] *Key Lab of Medical Molecular Virology of Ministries of Education and Health, School of Basic Medical Sciences & Shanghai Public Health Clinical Center, Fudan University, Shanghai, 200032, China;*

[d] *College of Life Sciences, Agricultural University of Hebei, Baoding, 071001, China;*

[e] *Center for Clinical Laboratory, Nanfang Hospital, Southern Medical University, Guangzhou, 510515, China;*

[f] *Lindsley F. Kimball Research Institute, New York Blood Center, New York, NY, 10065, USA*

[*] Corresponding author. School of Pharmaceutical Sciences, Southern Medical University, 1838 Guangzhou Avenue North, Guangzhou, Guangdong 510515, China.

E-mail addresses: li75lin@126.com (L. Li), liusw@smu.edu.cn (S. Liu).

[†] These authors contributed equally to this work.

Supplementary Figure legends

Supplementary Figure 1. **Pre-incubation with host cells or virus particles did not affect the antiviral activity of HP-API.** HP-API pre-incubated with **(A)** HIV-1_{IIIB} viral particles or **(B)** MT-2 cells at 37ºC for 30 min. After 4 days infection, TZM-bl cells were used as target cells and the luciferase activities were measured for detecting the inhibition of infection. Average values (± SD) were calculated from triplicate measurements; the data shown here represent one representative trial of three independent experiments.

A

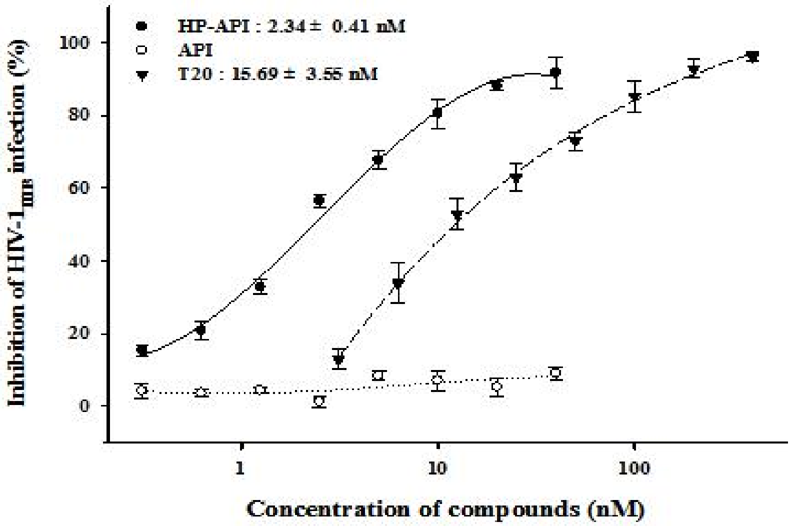

B

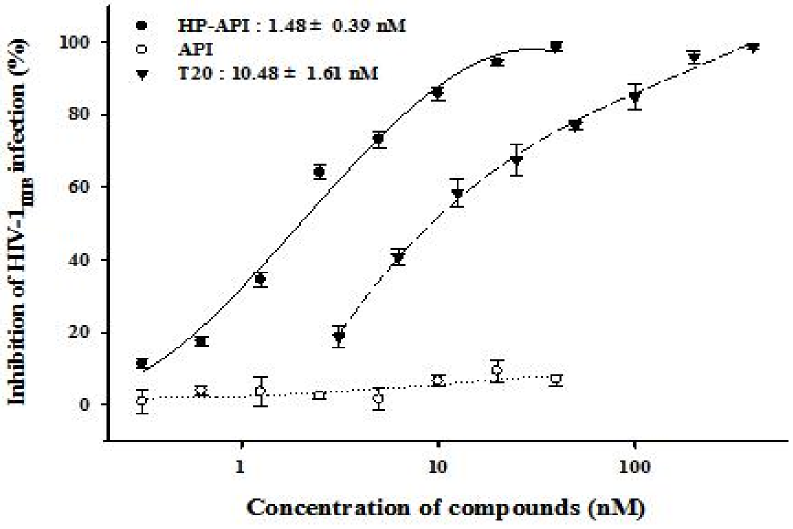

Supplementary Figure 1
